# Proceedings of the 2nd Congress on Evidence Based Mental Health: from research to clinical practice

**DOI:** 10.1186/s12991-018-0206-2

**Published:** 2018-08-28

## A1 Is bed rest helpful as an intervention in the management of severe anorexia nervosa in hospitals?

### Ali Ibrahim^1^, Agnes Ayton^2^

#### ^1^SLAM NHS Foundation Trust, London, UK; ^2^Oxford Health NHS Foundation Trust, Oxford, UK

##### **Correspondence:** Ali Ibrahim - ali.ibrahim@slam.nhs.uk

*Annals of General Psychiatry* 2018, **17(Suppl 1):**A1

**Background:** Bed rest has been part of treatment of severe anorexia nervosa in hospitals both in the UK and internationally. It is commonly used on medical and paediatric wards and both the adult and Junior MARSIPAN [1] guidelines recommend bed rest as part of nursing management of the physically compromised patient. However, recently there has been increasing awareness of the negative effect of bed rest in other patient populations.

The aim of this study was to review the evidence base of using bed rest as an intervention in the management of severe anorexia nervosa.

**Methods:** We searched on HDAS NICE website the following data bases: Medline, Pubmed, Embase, PsychInfo, Cinahl, Hmic, Amed, HBE, BNI including title and abstract for the following search terms: bed rest, anorexia nervosa, randomized controlled trial.

**Results:** 21,591 papers included the search term ‘bed rest’ and 56,131 ‘anorexia nervosa’. After exclusion of duplicates, only 17 papers included both topics. There were no randomised controlled trials. Negative physical consequences were described in a number of studies, and included lower heart rate, venous thrombosis, impaired bone turn over and increased risk of infection. Several papers showed that patients have a strong preference for less restrictive approaches. These are also less intensive in nursing time.

**Conclusions:** The evidence to support the use of bed rest in the management of hospitalised patients with severe anorexia nervosa is extremely limited. The risks associated with bed rest are significant, and include both physical and psychological harm. Given the clear risk of harm, it is difficult to recommend a randomised controlled trial on the subject, and the practice is best avoided altogether. Risks associated with initial refeeding can be managed in less restrictive manner.

**Reference**

1. Royal College of Psychiatrists *MARSIPAN: Management of Really Sick Patients with Anorexia Nervosa CR189*. 2014.

## A2 Assessing confinement environment stress using fNIRS

### Christina-Sylvia Andrea^1^, Yuichi Oi^2^, Yasuhito Hirai^2^, Shotaro Doki^3^, Yuh Ohtaki^1^, Daisuke Hori^4^, Tsukasa Takahashi^1^, Shin-ichiro Sasahara^2^, Tamaki Saito^2^, Ichiyo Matsuzaki^2,5^

#### ^1^Graduate School of Comprehensive Human Sciences, University of Tsukuba, Tsukuba, Japan; ^2^Faculty of medicine, University of Tsukuba, Tsukuba, Japan; ^3^Hospital Bando, Bando, Japan; ^4^Department of Environmental and Preventive Medicine, Kanazawa University Graduate School of Medical Sciences, Kanazawa, Japan; ^5^International Institute for Integrative Sleep Medicine, University of Tsukuba, Tsukuba, Japan

##### **Correspondence:** Christina-Sylvia Andrea

*Annals of General Psychiatry* 2018, **17(Suppl 1):**A2

**Background:** With human space exploration missions being a crucial focus of space programs, the number of astronauts (including Japanese astronauts) recruited for a long-term stay at the International Space Station (ISS) has increased. The confinement conditions that astronauts experience during the extended time periods spent in the same room with the same people when on mission, might present an important psychosocial stressor. However, an efficient methodology to measure the confinement-triggered psychological stress has yet to be established. Given that psychosocial stress is known to have negative effects on the frontal brain function, we attempted to assess stress-related index measurements of frontal brain function using functional near-infrared spectroscopy (fNIRS), a non-invasive method of monitoring brain activity by measuring the localized blood oxygenation levels of the brain, additionally to conventional stress-evaluation methods, under confinement conditions.

**Materials and methods:** To simulate the conditions experienced by astronauts during selection-examination and stay in the ISS, we used the “confinement environment adaptation facilities” of the Japan Aerospace Exploration Agency (JAXA), where research participants (n = 15, all adult males, divided into 2 groups) were confined for a period of 14 days during 2016. To measure the stress received in confinement, we monitored the participants’ frontal brain activation during a cognitive test (verbal fluency test [VFT]) using fNIRS. Conventional stress-evaluation methods, including the self-evaluation questionnaire of sense of coherence (SOC, 29-item scale), were also used to measure the perceived confinement-induced stress. Additional exercise intervention was applied during confinement to half of the participants (n = 8), who had to complete a 15-min exercise by aero bike every day (exercise was prohibited in the control group [n = 7]).

**Results:** fNIRS measurements showed a general decreasing tendency. SOC scores tended to increase. Interestingly, fNIRS measurements significantly differed between exercise intervention and control groups.

**Conclusions:** During confinement, the fNIRS measurements presented a general decrease, suggesting that fNIRS could possibly detect early signs of stress and depression. Though the confinement period was short, participants’ SOC had an upward trend, suggesting a strengthened sense of coherence due to the newly obtained experiences. Exercise had a positive effect towards maintaining good frontal brain function during confinement environment stress. fNIRS might be a useful tool to readily assess the psychosocial stress inflicted during confinement.

**Acknowledgements:** Ethical issues were reviewed and approved both from the University of Tsukuba Medical Ethics Committee (No. 1022) and the JAXA Ethical Review Board (1112001). We declare no conflict of interest. This study was supported by the JSPS Grant in Aid for scientific research (15H05941).

## A3 Deficiency of vitamin D levels in psychotic patients: can this be important?

### Maria Athanasiou

#### Self-employed psychiatrist, Alexandroupolis, Greece

##### **Correspondence:** Maria Athanasiou

*Annals of General Psychiatry* 2018, **17(Suppl 1):**A3

Vitamin D or calciferol, is a fat-soluble vitamin. It is known as a sunshine vitamin. It consists of a group of fat-soluble compounds (more than ten), the most important of which are, vitamin D_2_ or ergocalciferol and vitamin D_3_ or cholecalciferol. Vitamin D is produced endogenously in the skin from sun exposure sunlight (UVB) or obtained from food group’s supplements but is also widely used for the treatment and prevention of hypovitaminosis D (mainly D_2_). Vitamin D deficiency has been recognized as a pandemic with a myriad of health consequences. It has been associated with mental disorders and derives from decreased sun exposure but it is also associated with other factors.

Vitamin D, in its biologically active form 1, 25(OH) _2_ D, exhibits hormonal activity because it shares common features with steroid hormones. In the liver and kidneys, the biologically inactive circulating form of Vitamin D [25 (OH) D] bound to the vitamin D receptor (VDR), after 2 hydroxylations, is converted to its biologically active form 1,25-dihydroxyvitamin D [1,25(OH)_2_ D] which is responsible for the biological actions of vitamin D.

Over the past 25 years, a number of reports have suggested that Vitamin D has been expanded beyond its effects on calcium metabolism, and research evidence in the last 10 years indicates its function as a potent neurosteroid. In particular, vitamin D, with its most correct conceptual use as a hormone, has been thought to affect brain growth due to its antioxidant activity and its effect: a) on cell differentiation b) on cytokine regulation c) neurotransmitter synthesis d) expression of neurotrophic factor (BDNF) e) and expression of genes/proteins involved in differentiation, metabolism and neuronal structure.

In addition to its neuroprotective action, the growing literature of recent years associates its deficiency with psychotic disorders and manifestations. This association is reinforced by preclinical indications, which point out the role of vitamin D deficiency in dopaminergic activity as well as in neuronal differentiation and cerebral structure and function. Vitamin D is a new field of research, regarding psychotic disorders and manifestations. From the literature review is indicated that clinician should take into consideration vitamin D status.

## A4 New methods to assess the correlation between reward system and chronic fatigue in patients on chronic hemodialysis

### Michela Balconi^1,2^, Daniela De Filippis^1,2^, Laura Angioletti^1,2^, Maurizio Bossola^3^

#### ^1^Research Unit in Affective and Social Neuroscience, Catholic University of the Sacred Heart, Milan, Italy; ^2^Department of Psychology, Catholic University of the Sacred Heart, Milan, Italy; ^3^Hemodialysis Service, University Hospital Agostino Gemelli, Catholic University of the Sacred Heart, Rome, Italy

##### **Correspondence:** Michela Balconi - michela.balconi@unicatt.it

*Annals of General Psychiatry* 2018, **17(Suppl 1):**A4

**Background:** One of the most frequently observed clinical manifestation in patients with kidney disease on chronic hemodialysis (HD) treatment is fatigue, a complex symptom that deeply affects their quality of life [1]. Research suggested that fatigue is associated to chronic inflammation and disfunction of the basal ganglia, leading to an alteration of reward processes, in chronic patients [2]. Recently, there is a growing interest in finding new methods and perspectives to assess these dimensions in chronic populations. For this reason, this study aimed to examine the correlation between fatigue and motivational systems in HD patients.

**Materials and methods:** To assess the effect of fatigue on motivational and physical aspects, the Fatigue Severity Scale (FSS) was applied to a total of ninety-four hemodialysis patients. While the Behavioural Activation System (BAS) and Behavioural Inhibition System (BIS) Scale was administered to measure their mechanisms of reward, that predispose to activation or inhibition of action. Finally, State-Trait Anxiety Inventory (STAI-Y) and Beck Depression Inventory (BDI-II) were used to detect the presence of anxiety and depression.

**Results:** FSS score was significantly higher in patients with high BIS Z-score than in patients with low and medium BIS Z-score. Conversely, all BIS Z-score groups of patients showed similar BDI and STAI-Y1/Y2 scores. Finally, the correlation between BIS and FSS score was found to be significant.

**Conclusions:** Globally, these findings suggest a correlation between fatigue symptoms and the behavioral inhibition system that, within the motivational mechanisms, is related to the inhibition of action with possible consequences on patients’ engagement.

**References**

Bossola M, Di Stasio E, Antocicco M, Panico L, Pepe G, Tazza L. Fatigue is associated with increased risk of mortality in patients on chronic hemodialysis. Nephron. 2015; 130:113–118.
2. Bossola M, Di Stasio E, Giungi S, Rosa F, Tazza L. Fatigue Is Associated With Serum Interleukin-6 Levels and Symptoms of Depression in Patients on Chronic Hemodialysis. J Pain Symptom Manage. 2015;49:578–85.

## A5 Personality traits and neurophysiological correlates affect gambling behavior in Parkinson’s Disease

### Michela Balconi^1,2^, Laura Angioletti^1,2^, Chiara Siri^3^, Nicoletta Meucci^3^, Gianni Pezzoli^3^

#### ^1^Department of Psychology, Catholic University of the Sacred Heart, Milan, Italy; ^2^Research Unit in Affective and Social Neuroscience, Catholic University of the Sacred Heart, Milan, Italy; ^3^Parkinson Centre, ASST G. Pini-CTO, ex ICP, Milan, Italy

##### **Correspondence:** Michela Balconi - michela.balconi@unicatt.it

*Annals of General Psychiatry* 2018, **17(Suppl 1):**A5

**Background:** Personality traits, such as reward sensitivity and impulsivity, and dopaminergic treatment are crucial characteristics related to the development of gambling behavior in Parkinson’s Disease (PD) patients [1]. Pathological Gambling (PG) manifests as a persistent gambling behavior, characterized by dysfunctional decision-making, emotional impairment and high-risk behaviors [2, 3]. Thus, the role of personality components and prefrontal cortex activity in Parkinson’s patients with or without gambling were explored with a special focus on emotional and decision processes [4].

**Materials and methods:** Iowa Gambling Task (IGT) performance and hemodynamic cortical activity, measured by functional Near-Infrared Spectroscopy (fNIRS) were recorded in PD patients, divided into three groups according to their gambling status. Sample included PD patients with active gambling behavior (Parkinson’s Disease Gamblers, PDG); PD patients who remitted from PG (Parkinson’s Disease Non-Gamblers, PDNG); and a control group composed by patients with PD only.

**Results:** Findings highlighted that gambling behavior in Parkinson’s Disease Gamblers is strongly predictive of dysfunctional cognitive strategy; affecting anomalous cortical response with a left hemispheric unbalance in dorsal areas; and it is related to more reward sensitivity personality trait.

**Conclusions:** Overall, Parkinson’s Disease Gamblers patients differed from Parkinson’s Disease Non-Gamblers and control group from both behavioral and brain response to decision-making, showing a pathological condition related to emotional and cognitive aspects.

**References**

1. Brewer JA, Potenza MN. The neurobiology and genetics of impulse control disorders: Relationships to drug addictions. Biochem Pharmacol. 2008; 75:63–75.
2. Balconi M, Finocchiaro R. Decisional impairments in cocaine addiction, reward bias, and cortical oscillation “unbalance.” Neuropsychiatr Dis Treat. 2015; 11:777–86.
3. Balconi M, Finocchiaro R, Campanella S. Reward Sensitivity, Decisional Bias, and Metacognitive Deficits in Cocaine Drug Addiction. J Addict Med. 2014; 8:399–406.
4. Balconi M, Grippa E, Vanutelli ME. What hemodynamic (fNIRS), electrophysiological (EEG) and autonomic integrated measures can tell us about emotional processing. Brain Cogn. 2015; 95:67–76.

## A6 Problems and pitfalls in the clinical assessment process for the diagnosis of specific learning difficulties (dyslexia) in Greece

### Eleni Bonti, Sophia Vavetsi, Vasiiki Loizou

#### Department of 1st Psychiatric Clinic of ‘Papageorgiou’ General Hospital of Thessaloniki, Aristotle University of Thessaloniki, Thessaloniki, Ring Road Thessaloniki, N. Efkarpia, GR-54603, Greece

##### **Correspondence:** Eleni Bonti - bonti@auth.gr; elina.bonti@gmail.com

*Annals of General Psychiatry* 2018, **17(Suppl 1):**A6

**Background: **Introduction ‘Specific Learning Difficulties’ (SLD) is a general term, (often used as a synonym for Dyslexia), used to describe an extremely heterogeneous population of different age groups who struggle with several areas of learning. The heterogeneity of SLD along with several other factors often lead to a number of diagnostic and intervention pitfalls and errors [1].

**Materials and Methods:** Based on our everyday clinical practice at the State Certified Diagnostic Center for Learning Difficulties of ‘Papageorgiou’ General Hospital of Thessaloniki and the constant challenge of coming up with a valid clinical assessment for people with possible SLD and taking into account the relevant research, we decided to review the most important key points concerning these challenges.

**Results:** The numerous definitions for describing SLD (or Dyslexia), the two different models of pathogenesis, as well as the different subtypes of SLD have led to a number of diagnostic methods. As a result, multiple issues regarding diagnostic validity and reliability occur. The non-standardized assessment tools mainly used by clinicians in Greece due to the absence of a common, standardized diagnostic tool for the diagnosis of SLD raise questions concerning the validity and reliability of diagnosis. The inadequate staffing of diagnostic services in Greece, the lack of sufficiently trained staff, and the incomplete relevant legislation regarding the SLD population in Greece, often lead to diagnostic errors and pitfalls and to inappropriate intervention practices. Assessment procedures in Greece often lack parts of important information regarding the developmental, educational and social record of the person being assessed, thus, providing limited information required for a safer diagnosis. The IQ tests used for an SLD diagnosis, fail to provide the necessary information for planning a successful individual intervention program. Differential Diagnosis is also a big challenge for the Greek diagnostic services, given their limitations mentioned above. Poor information, ignorance, or denial factors of parents and teachers in Greece often prevent the early detection of the ‘risk’ signs and characteristics of possible SLD among preschool age children.

**Conclusions:** Diagnosis plays a decisive role in the configuration of individual identity and the quality living of people with SLD and their families. The possible errors and pitfalls that might occur during an assessment process may have immense consequences to the learner with SLD, both at an educational, as well as at a psychological and social level. A wrong or an invalid diagnosis may convert school years to a day to day ‘nightmare’ and adult life to an ongoing race. Therefore, the diagnostic procedures carried out for students with possible SLD (or other types of learning difficulties), especially in Greece, should be very carefully designed, using a combination of appropriate tools and should be carried out by a multi-professional team of different, well-trained specialists who will be able to consider all the possible pitfalls that might occur during this challenging and dynamic procedure.

## A7 Examining the role of repetitive transcranial magnetic stimulation (RTMS) for the treatment of obsessive–compulsive disorder (OCD)—a meta-analysis

### Vlasios Brakoulias^1^, Simone Rehn^1,2^

#### ^1^Discipline of Psychiatry, University of Sydney, Sydney Medical School Nepean, Sydney, Australia; ^2^School of Psychology, University of Sydney, Sydney, Australia

##### **Correspondence:** Vlasios Brakoulias - vbrakoulias@bigpond.com

*Annals of General Psychiatry* 2018, **17(Suppl 1):**A7

**Background:** There have been several randomised controlled trials attempting to determine whether repetitive transcranial magnetic stimulation (rTMS) can help treat OCD. Some have reported that rTMS is effective, whereas others have not. To present and discuss a meta-analysis of randomized controlled trials to date with regards to the treatment of OCD with rTMS with a specific aim of attempting to determine the factors that may be associated with a positive treatment response.

**Materials and methods:** After a systematic literature review, results of treatment trials using rTMS for OCD were subjected to meta-analysis. In order to determine specific factors that may have influenced success rates, studies were analysed according to high or low frequency rTMS, anatomical sites of stimulation and length of follow-up.

**Results:** There have been 18 randomised controlled trials attempting to determine whether repetitive transcranial stimulation (rTMS) can help treat OCD. Overall, they showed a modest effect in reducing Y-BOCS scores with an odd ratio of treatment response of 0.23 (95% CI 0.12–0.43, p < 0.001). Low frequency (< 1 Hz) rTMS appeared more effective than high frequency (> 5 Hz) rTMS. Stimulation of the supplementary motor area appeared more effective than stimulation over the dorsolateral prefrontal cortex. Results were better at 3 months follow-up than at 4 weeks follow-up. There was no publication bias.

**Conclusions:** The use of rTMS as an adjunctive treatment for OCD requires further investigation with this meta-analysis indicating that the low frequency rTMS and stimulation of the supplementary motor area might be more effective strategies. The study also suggests that a longer follow up period, e.g. 3 months after treatment might be better for patients with OCD.

## A8 Young smokers’ emotional self-perceived voice level: in depth data analysis of administered voice handicap index

### Evangelia I. Kosma^1,2^, Dionysios Tafiadis^3^, Spyridon K. Chronopoulos^4,5^

#### ^1^Faculty of Medicine, School of Health Sciences, University of Thessaly, Biopolis, Larissa, Greece; ^2^Psychologist, Private Practice, Ioannina, Greece; ^3^Department of Speech & Language Therapy, Technological Educational Institute of Epirus, Ioannina, Greece; ^4^Department of Informatics and Telecommunications Engineering, University of Western Macedonia, Kozani, Greece; ^5^Department of Computer Engineering, Technological Educational Institute of Epirus, Arta, Greece

##### **Correspondence:** Spyridon K. Chronopoulos - schronopoulos@uowm.gr; spychro@gmail.com

*Annals of General Psychiatry* 2018, **17(Suppl 1):**A8

**Background:** Several studies have shown that smoking is the primary candidate for provoking voice disorders in adults. This unwanted condition has unfortunately other additional unwanted effects. One strong effect which is the result of this study, is relevant to psychological affected aspect of subjects which smoked [1–4]. For the purpose of conducting this research the Voice Handicap Index (VHI) emotional domain of items was administered to subjects. This domain included 10 items relevant to checking the psychological and psychosocial condition of subjects.

**Materials and methods:** Two hundred and fifty participants (120 females and 130 males) were recruited. This sample constituted of 128 smokers (60 females and 68 male) and 122 nonsmokers (60 females and 62 male). The participants, exhibiting any voice complaint or other medical condition along with the presence of prohibited environmental conditions which could affect their voice, were excluded from this study [5–6]. “Non-smokers” were considered the subjects who had never smoked prior to this research. All participants filled in the translated version of Voice Evaluation Template (VET) and the standardized Hellenic version of Voice Handicap Index (VHI).

**Results:** Smokers had a significant higher VHI-E (U = 1799.000, P < .001) compared to non-smokers. Similarly, male smokers’ results exhibited significant differences compared to non-smokers’ VHI-E (U = 442.500, P < .001). Similarly, female smokers showed higher emotional score in comparison to non-smokers (U = 426.000, P = .000). The smokers’ subgroup (in all comparisons) exhibited the higher achieved scores.

**Conclusions:** It seems (based on VHI’s items) that the smokers in comparison to non-smokers are in greater stress condition and discomfort which in turn pushes their self-esteem in lower levels while it increases their feelings of shame. Moreover it appears that smokers receive the irritated behavior of others as they do not comprehend the voice problem of smokers [1–4]. Moreover the others probably do not understand the smokers’ condition and eventually do not express empathy towards them. The smokers’ low self-esteem in conjunction to being not accepted (social exclusion) by the non-smokers affect their everyday life while reducing their social contacting. In turn, this condition increases the time of isolation and consequently the risk of imminent depression and anxiety disorders.

**References**

1. Tafiadis D, Chronopoulos S K, Kosma E I et al. Using Receiver Operating Characteristic Curve to Define the Cutoff Points of Voice Handicap Index Applied to Young Adult Male Smokers. Journal of Voice. 2018;32(4):443–448. 10.1016/j.jvoice.2017.06.007
2. Tafiadis D, Kosma E I, Chronopoulos S K et al. Voice Handicap Index and Interpretation of the Cutoff Points Using Receiver Operating Characteristic Curve as Screening for Young Adult Female Smokers. Journal of Voice. 2018;32(1):64–69. 10.1016/j.jvoice.2017.03.009 
3. Tafiadis D, Chronopoulos S, Siafaka V et al. Comparison of Voice Handicap Index Scores Between Female Students of Speech Therapy and Other Health Professions. Journal of Voice. 2017;31(5):583–588. 10.1016/j.jvoice.2017.01.013
4. Tafiadis D, Kosma E, Chronopoulos S et al. Acoustic and Perceived Measurements Certifying Tango as Voice Treatment Method. Journal of Voice. 2018;32(2):256.e13–256.e24. 10.1016/j.jvoice.2017.05.016
5. Tafiadis D, Helidoni M, Chronopoulos S et al. Preliminary Receiver Operating Characteristic Analysis on Voice Handicap Index of Laryngeal Inflammation in Greek Patients. International Journal of Otolaryngology and Head & Neck Surgery. 2018;07(03):115–131. 10.4236/ijohns.2018.73014
6. Tafiadis D, Kosma E, Chronopoulos S, Voniati L, Ziavra N. A Preliminary Receiver Operating Characteristic Analysis on Voice Handicap Index Results of the Greek Voice-Disordered Patients. International Journal of Otolaryngology and Head & Neck Surgery. 2018;07(03):98–114. 10.4236/ijohns.2018.73013

**Ethics Approval:** The study was approved by the Ethical Committee of Department of Speech Language Therapy (School of Health and Welfare Professions) TEI of Epirus

**Consent to publish:** Informed consent to publish has been obtained from each participant.

## A9 Extensive data analysis on the tango voice treatment method relevant to its positive emotional effects using voice handicap index

### Evangelia I. Kosma^1,2^, Dionysios Tafiadis^3^, Spyridon K. Chronopoulos^4,5^

#### ^1^Faculty of Medicine, School of Health Sciences, University of Thessaly, Biopolis, Larissa, Greece; ^2^Psychologist, Private Practice, Ioannina, Greece; ^3^Department of Speech & Language Therapy, Technological Educational Institute of Epirus, Ioannina, Greece; ^4^Department of Informatics and Telecommunications Engineering, University of Western Macedonia, Kozani, Greece; ^5^Department of Computer Engineering, Technological Educational Institute of Epirus, Arta, Greece

##### **Correspondence:** Evangelia I. Kosma - ekosma@med.uth.gr; evikosma@gmail.com

*Annals of General Psychiatry* 2018, **17(Suppl 1):**A9

**Background:** Various therapies of voice disorders exist such as the kind of invasive treatments. On the contrary, a significant non-invasional method, demanding only the physical exercise of the subject, is Tango Voice Treatment Method [1]. Its beneficial results constitute this voice therapy as a standalone procedure even without hydration (not a strong factor). This research prolongs the beneficial results of this method by checking the emotional effects by administering Voice Handicap Index (VHI) [2–3]. These effects are directly associated with voice disorders and they include stress, emotional, or personality factors. It is well-known that emotional distress maybe related to voice disorders through a vicious cycle without distinguishing between them the cause and the result [4].

**Materials and methods:** In this study fifty-two adults (22 dancers and 30 non-dancers) were recruited. All participants had no former history of ENT (Ear-Nose-Throat) disorders or had former history of at least 2 weeks before enrolment while the environmental living, working or physical conditions were not prohibited relevant to affecting their voice condition. Tango dancers were considered the individuals with at least two dance sessions per week for 1 year. As for the control group, its participants never danced. All subjects filled in the Voice Evaluation Template (VET) and the Hellenic Voice Handicap Index (VHI) [5–7].

**Results:** Non-dancers had a significant higher VHI-Emotional score (U = 192.500, P = .001) compared to tango dancers. Similarly, the female non-dancers exhibited significant differences compared to dancers VHI-E (U = 38.000, P < .001). Likewise, by comparing all divided subgroups according to gender, statistical significant changes were calculated relevant to VHI-Emotional scores (H(3) = 7.657, P < .050). Also, the previous results were similar to the case of male non-dancers who had higher emotional score in comparison to tango dancers (U = 55.500, NS) and this result was not statistically significant. In general, the non-dancer subgroup (in all comparisons) exhibited the higher achieved scores.

**Conclusions:** The Tango dancers in comparison to non-dancers exhibited better emotional condition which could be consisted of three significant beneficial categories. The first category is related to lower stress levels, lower discomfort and shame during the communication procedure with others. The second beneficial category is related to a more positive behavior from others towards Tango dancers. The third beneficial category is the enhanced social contacting in conjunction to the reduced anxiety in Tango dancers’ voice. In conclusion, the overall benefits are prominent for the emotional voice status of the subjects under Tango Voice Treatment Method.

**References**

1. Tafiadis D, Kosma E, Chronopoulos S et al. Acoustic and Perceived Measurements Certifying Tango as Voice Treatment Method. Journal of Voice. 2018;32(2):256.e13–256.e24. 10.1016/j.jvoice.2017.05.016
2. Tafiadis D, Chronopoulos S K, Kosma E I et al. Using Receiver Operating Characteristic Curve to Define the Cutoff Points of Voice Handicap Index Applied to Young Adult Male Smokers. Journal of Voice. 2018;32(4):443–448. 10.1016/j.jvoice.2017.06.007 
3. Tafiadis D, Kosma E I, Chronopoulos S K et al. Voice Handicap Index and Interpretation of the Cutoff Points Using Receiver Operating Characteristic Curve as Screening for Young Adult Female Smokers. Journal of Voice. 2018;32(1):64–69. 10.1016/j.jvoice.2017.03.009 
4. Dietrich M, Verdolini Abbott K, Gartner-Schmidt J, Rosen C. The Frequency of Perceived Stress, Anxiety, and Depression in Patients with Common Pathologies Affecting Voice. Journal of Voice. 2008;22(4):472–488. 10.1016/j.jvoice.2006.08.007
5. Tafiadis D, Chronopoulos S, Siafaka V et al. Comparison of Voice Handicap Index Scores Between Female Students of Speech Therapy and Other Health Professions. Journal of Voice. 2017;31(5):583–588. 10.1016/j.jvoice.2017.01.013
6. Tafiadis D, Kosma E, Chronopoulos S, Voniati L, Ziavra N. A Preliminary Receiver Operating Characteristic Analysis on Voice Handicap Index Results of the Greek Voice-Disordered Patients. International Journal of Otolaryngology and Head & Neck Surgery. 2018;07(03):98–114. 10.4236/ijohns.2018.73013
7. Tafiadis D, Helidoni M, Chronopoulos S et al. Preliminary Receiver Operating Characteristic Analysis on Voice Handicap Index of Laryngeal Inflammation in Greek Patients. International Journal of Otolaryngology and Head & Neck Surgery. 2018;07(03):115–131. 10.4236/ijohns.2018.73014

**Ethics Approval:** The study was approved by the Ethical Committee of Department of Speech Language Therapy (School of Health and Welfare Professions) TEI of Epirus

**Consent to publish:** Informed consent to publish has been obtained from each participant.

## A10 A proposed research protocol for investigating the beneficial effects of green office buildings on their workers, with the use of data analysis

### Evangelia I. Kosma^1,2^, Spyridon K. Chronopoulos^3,4^, Dionysios Tafiadis^5^, Vasilis Christofilakis^3^, Panos Kostarakis^3^

#### ^1^Faculty of Medicine, School of Health Sciences, University of Thessaly, Biopolis, Larissa, Greece; ^2^Psychologist, Private Practice, Ioannina, Greece; ^3^Physics Department, Electronics-Telecommunications and Applications Laboratory, University of Ioannina, Ioannina, Greece; ^4^Department of Informatics and Telecommunications Engineering, University of Western Macedonia, Kozani, Greece; ^5^Department of Speech & Language Therapy, Technological Educational Institute of Epirus, Ioannina, Greece

##### **Correspondence:** Spyridon K. Chronopoulos - schronopoulos@uowm.gr; spychro@gmail.com

*Annals of General Psychiatry* 2018, **17(Suppl 1):**A10

**Background:** In recent years, the usefulness of Green Technology along with the implementation of advanced wireless technologies led scientists towards investigating the effects of this advanced Green Technology to Human Resources. A large number of surveys have investigated the impact, of working conditions inside conventional buildings, on health, productivity, and job satisfaction. This research “stream” have led to the green revolution. Insufficient Indoor Environmental Quality (IEQ) has been shown to be the cause or the burden of a pre-existing physical illness such as a respiratory problem (asthma, infections and allergic rhinitis), musculoskeletal problems, and tendency towards psychological effects (cognitive impairment, depression, anxiety, vocal disorders) [1–3]. Labor absence and productivity decrease are related to the aforementioned problems, while the lack of job satisfaction is often referred to as a consequence. Consequently this research is intended to discover the beneficial effects to Greek Human Resources from utilizing Green Office Buildings.

**Materials and methods:** The following proposed research protocol is comprised of 30 employees working in a conventional building located in a Greek area. The sample does not include people such as smokers, asthmatics, people with known depression or anxiety disorder, and people with chronic illnesses. The first phase of the survey will be conducted by studying the employees during their work hours in a conventional building for a period of 3 weeks, while in the second phase, the workers will move in a green building to work for the same number of weeks. The following tools will be used such as demographics acquisition which will be filled in through a new questionnaire (applicable only in the first stage), the physical condition of employees that will be assessed using the Sick Building Syndrome (SBS) questions, the psychological condition with the use of Warwick-Edinburgh Mental Well-Being Scale (WEMWBS), the job satisfaction with the Minnesota Satisfaction Questionnaire-MSQ and the Occupational Stress Inventory-Revised/OSI-R. Employees’ productivity will be measured with the use of produced reports by their supervisors in both stages of the survey. In addition, it is proposed to use a sensor package including a Netatmo Weather Station and a portable measuring device such as Smart Watch. The measurement will include at least skin temperature and conductance, heart pulse, acceleration and oxygen saturation, while the Netatmo will measure temperature, humidity, CO2 concentrations and sound levels in dB on a regular basis.

**Results:** The methods that will be used will include probably t-tests in order to examine differences inside the worker’ group of Green Building and the control group (working in a typical building). As the sample will be of small size, Kruskal–Wallis tests will be employed as they are the most appropriate for these occasions. Additionally, Mann–Whitney U tests will compare the two groups relevant to a same collection data type. It is expected that employees working in a green building will exhibit increased productivity, reduced work-absences, and at the same time they will improve any physical problems or eliminate psychological problems which emerge from the working conditions inside a conventional building.

**Conclusions:** Alteration of working environmental conditions can affect positively the physical and psychological health of workers. The later has been shown by various surveys which they have compared the green buildings with conventional buildings. This study wants to confirm the aforementioned in the case of our already developed protocol named as Reduced Ecological Footprints of Modern Facilities (REFF) [4].

**References**

1. Thatcher A, Milner K. Changes in productivity, psychological wellbeing and physical wellbeing from working in a green building. Work. 2014;49(3):381–393.
2. Tafiadis D, Chronopoulos S K, Kosma E I et al. Using Receiver Operating Characteristic Curve to Define the Cutoff Points of Voice Handicap Index Applied to Young Adult Male Smokers. Journal of Voice. 2018;32(4):443–448. 10.1016/j.jvoice.2017.06.007 
3. Tafiadis D, Kosma E I, Chronopoulos S K et al. Voice Handicap Index and Interpretation of the Cutoff Points Using Receiver Operating Characteristic Curve as Screening for Young Adult Female Smokers. Journal of Voice. 2018;32(1):64–69. 10.1016/j.jvoice.2017.03.009 
4. Chronopoulos S, Kosma E, Tafiadis D et al. Reduced Ecological Footprints of Modern Facilities Introducing the Implementation of Advanced Wireless Technologies, and Human Resources’ Benefits. Communications and Network. 2018;10(01):11–29. 10.4236/cn.2018.101002

**Ethics Approval:** The study was approved by the Ethical Committee of Department of Speech Language Therapy (School of Health and Welfare Professions) TEI of Epirus

**Consent to publish:** Informed consent to publish has been obtained from each participant.

## A11 Preliminary design of a Smart Logic, Electronic and Green Public Health Questionnaire (SLE-GPHQ) for investigating the proper compatibility between people and green facilities

### Spyridon K. Chronopoulos^1,2^, Evangelia I. Kosma^3,4^, Dionysios Tafiadis^5^, Periklis Papadopoulos^6^, Ngoc-Tu Nguyen^7^, Pantelis Angelidis^2^, Panos Kostarakis^1^

#### ^1^Physics Department, Electronics-Telecommunications and Applications Laboratory, University of Ioannina, Ioannina, Greece; ^2^Department of Informatics and Telecommunications Engineering, University of Western Macedonia, Kozani, Greece; ^3^Faculty of Medicine, School of Health Sciences, University of Thessaly, Biopolis, Larissa, Greece; ^4^Psychologist, Private Practice, Ioannina, Greece; ^5^Department of Speech & Language Therapy, Technological Educational Institute of Epirus, Ioannina, Greece; ^6^Physics Department, University of Ioannina, Ioannina, Greece; ^7^Department of Computer Science & Engineering, University of Minnesota, Twin Cities, Minneapolis, MN 55455, USA

##### **Correspondence:** Evangelia I. Kosma - ekosma@med.uth.gr; evikosma@gmail.com

*Annals of General Psychiatry* 2018, **17(Suppl 1):**A11

**Background:** The new era of the continuous deployment of wireless and sensory technology in conjunction to the rapid expansion of Green Buildings’ has led to a point of developing and enforcing specific rules and conditions. These conditions depend on the proper matching of Peoples’ temperament and Green Smart Buildings. In parallel, the rules are relevant to the constructed Protocols for accomplishing the most efficient result relevant to the proper implementation of Green Buildings (LEED—Leadership in Energy and Environmental Design). This preliminary work is intended to find the best match between Habitants (or Workers) and Green Buildings based on an already proposed Protocol named as REFF (Reduced Ecological Footprints of Modern Facilities) [1].

**Materials and methods:** This work will firstly focus on the needed conditions in order to obtain a health green environment which should be consisted of nine fundamental elements which are proposed in [2]. These will include high quality of air quality (Indoor Air Quality—IAQ), the use of proper ventilation, thermal health, high water quality, minimized moisture, safety and security, reduced noise, proper lighting (frequencies below 100 Hz are prohibited) and absence of dust. Secondly our already constructed protocol of REFF [1] will be applied and readjusted relevant to the aforementioned. Then, 40 people will be tested into various green environments inside the same LEED Building (for 2 weeks) while using various personality tests [3] and productivity measurements in order to construct a map of Green Environment to Character Correspondence named as GEtoBCC. After this procedure, an electronic questionnaire will be constructed which will primarily be constituted of nine parts. Each part will correspond to one of the nine fundamental elements in conjunction to REFF. The Smart Logic will be based on Python, HTML and/or MATLAB code in order to rearrange the questionnaire relevant to each personality. This will conclude into better foreseeing the proper environment for each Habitant/Worker.

**Results:** Twenty people (10 men and 10 women) along with a control group of twenty people (10 men and 10 women) will be tested under different green environmental conditions. The control group will correspond to people working in a typical conventional building. In general, smokers will be excluded from the sample [4–6] and thus it will probably be split into independent subgroups relevant to character correspondence [3]. Consequently ANOVA will be employed in order to compare the unrelated “character” groups. In turn, and if the distribution of different populations is normal, a t-test will be used to compare each population with the mean of the control group. Then, Kruskal–Wallis tests will be conducted as they are the most appropriate for such small samples exhibiting exceptional results. Also, in the occasion of skewed variables, Mann–Whitney U tests will compare the different “character” groups relevant to a same data type collection. It is expected that the different “character” groups will interact differently inside altered green environments. This will help scientists, ranging from engineers to psychologists and other clinicians, to find and determine a viable solution relevant to the proper correspondence of population to various types of green buildings.

**Conclusions:** This work is focused on the construction of a new questionnaire named as SLEGPHQ in conjunction to the rules proposed by REFF protocol [1]. This electronic questionnaire will be self-adaptive to different types of answers and consequently will lead to the proper estimation of the appropriate “character” corresponding to the compatible LEED Building. If the aforementioned tactics will be employed in the case of people being moved at LEED Buildings then the final resulting populations of these facilities will be cohesive and will act towards the best possible maintenance of the new micro-environment.

**References**

1. Chronopoulos S, Kosma E, Tafiadis D et al. Reduced Ecological Footprints of Modern Facilities Introducing the Implementation of Advanced Wireless Technologies, and Human Resources’ Benefits. Communications and Network. 2018;10(01):11–29. 10.4236/cn.2018.101002
2. Cedeño-Laurent J, Williams A, MacNaughton P et al. Building Evidence for Health: Green Buildings, Current Science, and Future Challenges. Annu Rev Public Health. 2018;39(1):291–308. 10.1146/annurev-publhealth-031816-044420
3. Loehlin J, Martin N. Personality types: A twin study. Pers Individ Dif. 2018;122:99–103. 10.1016/j.paid.2017.10.012
4. Tafiadis D, Chronopoulos S K, Kosma E I et al. Using Receiver Operating Characteristic Curve to Define the Cutoff Points of Voice Handicap Index Applied to Young Adult Male Smokers. Journal of Voice. 2018;32(4):443–448. 10.1016/j.jvoice.2017.06.007 
5. Tafiadis D, Kosma E I, Chronopoulos S K et al. Voice Handicap Index and Interpretation of the Cutoff Points Using Receiver Operating Characteristic Curve as Screening for Young Adult Female Smokers. Journal of Voice. 2018;32(1):64–69. 10.1016/j.jvoice.2017.03.009
6. Tafiadis D, Chronopoulos S, Siafaka V et al. Comparison of Voice Handicap Index Scores Between Female Students of Speech Therapy and Other Health Professions. Journal of Voice. 2017;31(5):583–588. 10.1016/j.jvoice.2017.01.013

**Ethics Approval:** The study was approved by the Ethical Committee of Department of Speech Language Therapy (School of Health and Welfare Professions) TEI of Epirus

**Consent to publish:** Informed consent to publish has been obtained from each participant.

## A12 The impact of green systems and signals on the health of green residences’ habitants

### Evangelia I. Kosma^1,2^, Spyridon K. Chronopoulos^3,4^, Dionysios Tafiadis^5^, Periklis Papadopoulos^6^, Ngoc-Tu Nguyen^7^, Vasilis Christofilakis^3^, Panos Kostarakis^3^

#### ^1^Faculty of Medicine, School of Health Sciences, University of Thessaly, Biopolis, Larissa, Greece; ^2^Psychologist, Private Practice, Ioannina, Greece; ^3^Physics Department, Electronics-Telecommunications and Applications Laboratory, University of Ioannina, Ioannina, Greece; ^4^Department of Informatics and Telecommunications Engineering, University of Western Macedonia, Kozani, Greece; ^5^Department of Speech & Language Therapy, Technological Educational Institute of Epirus, Ioannina, Greece; ^6^Physics Department, University of Ioannina, Ioannina, Greece; ^7^Department of Computer Science & Engineering, University of Minnesota, Twin Cities, Minneapolis, MN 55455, USA

##### **Correspondence:** Spyridon K. Chronopoulos - schronopoulos@uowm.gr; spychro@gmail.com

*Annals of General Psychiatry* 2018, **17(Suppl 1):**A12

**Background:** The promotion of green growth has been a strategy for developed countries by improving the energy performance of residences. This tendency will help towards overcoming the continuously increasing greenhouse effect because the contribution of typical residences to the production of air pollutants is extremely high. Housing conditions (indoor air quality, building and furnishing materials, noise, humidity, temperature, lighting, dust, tobacco, chemical cleaners) have been a forming factor of the physical and mental health of residents. The proposed survey aims to investigate the impact of Green Residences on the physical and psychological health of their occupants. The design of these residences will include REFF Protocol [1]. This investigation will be focused on correlating the Indoor Environmental Quality (IEQ) with the residents’ health and quality of life.

**Materials and methods:** The sample of the proposed research will include 30 families (adults with children) living in conventional houses and 30 families living at least 1 year in green houses in the Greek area. Adults and children with chronic illnesses such as asthma, chronic obstructive pulmonary disease, allergies, mental health problems, and Sick Building Syndrome (SBS) symptoms will be included in the survey to assess their burden or health improvement according to their living conditions over the past year. All kind of smokers and people with voice disorders will be excluded from this research [2–6]. In the proposed research protocol, the following questionnaires will be included: The demographics of the participants will be filled in an impromptu questionnaire, the physical condition will be evaluated with the Sick Building Syndrome Questions and additionally will be recorded in a self-improvised questionnaire concerning the past year, the Psychological condition will be recorded with the Warwick-Edinburgh Mental Well-Being Scale (WEMWBS) and the quality of life with the SF-36 Health Profile.

**Results:** Green residences are expected to greatly outperform conventional ones over time as they offer improved living conditions, superior electronic micro-environmental systems and at the same time low radiation sources (e.g. Wireless devices with adaptable and extremely low energy transmitted signals). In particular, we predict that residents of green houses will experience lower incidence of symptoms such as sick building syndrome (dizziness, headaches, nausea, coughing, tiredness, nosebleeds, breathing problems, blurred vision, wheezing, sneezing, ear infection, skin rashes). Additionally, it is quite likely that chronic conditions, such as asthma (in adults and children), chronic obstructive pulmonary disease, allergies and cardiovascular problems, will show a remission in the symptoms. In addition, residents of green houses will outperform in mental and neurological health [7–9]. The corresponding levels are intertwined with the quality of life’s index which is expected to exhibit a high score.

**Conclusions:** Green residences sustain living conditions which promote the health of the beneficiaries and, in particular, appear to have a positive effect on the physical and mental health of their occupants, while improving their quality of life.

**References**

1. Chronopoulos S, Kosma E, Tafiadis D et al. Reduced Ecological Footprints of Modern Facilities Introducing the Implementation of Advanced Wireless Technologies, and Human Resources’ Benefits. Communications and Network. 2018;10(01):11–29. 10.4236/cn.2018.101002
2. Tafiadis D, Chronopoulos S K, Kosma E I et al. Using Receiver Operating Characteristic Curve to Define the Cutoff Points of Voice Handicap Index Applied to Young Adult Male Smokers. Journal of Voice. 2018;32(4):443–448. 10.1016/j.jvoice.2017.06.007 
3. Tafiadis D, Kosma E I, Chronopoulos S K et al. Voice Handicap Index and Interpretation of the Cutoff Points Using Receiver Operating Characteristic Curve as Screening for Young Adult Female Smokers. Journal of Voice. 2018;32(1):64–69. 10.1016/j.jvoice.2017.03.009 
4. Tafiadis D, Chronopoulos S, Siafaka V et al. Comparison of Voice Handicap Index Scores Between Female Students of Speech Therapy and Other Health Professions. Journal of Voice. 2017;31(5):583–588. 10.1016/j.jvoice.2017.01.013
5. Tafiadis D, Helidoni M, Chronopoulos S et al. Preliminary Receiver Operating Characteristic Analysis on Voice Handicap Index of Laryngeal Inflammation in Greek Patients. International Journal of Otolaryngology and Head & Neck Surgery. 2018;07(03):115–131. 10.4236/ijohns.2018.73014
6. Tafiadis D, Kosma E, Chronopoulos S, Voniati L, Ziavra N. A Preliminary Receiver Operating Characteristic Analysis on Voice Handicap Index Results of the Greek Voice-Disordered Patients. International Journal of Otolaryngology and Head & Neck Surgery. 2018;07(03):98–114. 10.4236/ijohns.2018.73013
7. Voniati L. School Based Speech and Language Therapists’ Perceptions of MLU-w and Its Use. Science Journal of Education. 2015; 3(6):119–125. 10.11648/j.sjedu.20150306.11
8. Voniati L. Christopoulou M. Developing an Augmentative and Alternative Communication System for a Child with Autism Spectrum Disorder: A Case Study, American Journal of Health Research. 2017; 1(2): 58–62. 10.11648/j.ijcsd.20170102.15
9. Voniati L. Charalambous, I. Rett syndrome: Clinical recognition, communication skills and therapeutic intervention, Archives of Hellenic Medicine. 2018; 35(2): 188–197.

1. 

**Ethics Approval:** The study was approved by the Ethical Committee of Department of Speech Language Therapy (School of Health and Welfare Professions) TEI of Epirus

**Consent to publish:** Informed consent to publish has been obtained from each participant.

## A13 A preliminary proposed scheme for Smart Talking Green Environment (STaGE) using empathetic voice signal rules for implementation into Green Systems

### Spyridon K. Chronopoulos^1,2^, Evangelia I. Kosma^3,4^, Dionysios Tafiadis^5^, Anastasios Skrivanos^6^, Ngoc-Tu Nguyen^7^, Kostas P Peppas^6^, Periklis Papadopoulos^8^, Pantelis Angelidis^2^, Panos Kostarakis^1^

#### ^1^Physics Department, Electronics-Telecommunications and Applications Laboratory, University of Ioannina, Ioannina, Greece; ^2^Department of Informatics and Telecommunications Engineering, University of Western Macedonia, Kozani, Greece; ^3^Faculty of Medicine, School of Health Sciences, University of Thessaly, Biopolis, Larissa, Greece; ^4^Psychologist, Private Practice, Ioannina, Greece; ^5^Department of Speech & Language Therapy, Technological Educational Institute of Epirus, Ioannina, Greece; ^6^Department of Informatics and Telecommunications, University of Peloponnese, Akadimaikou G. K. Vlahou, Tripoli, Greece; ^7^Department of Computer Science & Engineering, University of Minnesota, Twin Cities, Minneapolis, MN 55455, USA; ^8^Physics Department, University of Ioannina, Ioannina, Greece

##### **Correspondence:** Evangelia I. Kosma - ekosma@med.uth.gr; evikosma@gmail.com

*Annals of General Psychiatry* 2018, **17(Suppl 1):**A13

**Background:** Green residences are known for their innovative character towards living conditions and low energy emissions. Their reduced ecological footprints design is not only limited to aforementioned but also include the construction of better environmental spaces and conditions even through transforming conventional buildings. Moreover, the psychological factor plays an important role in redefying and updating the already developed green logic. If we concentrate into implementing an evolutionary smart talking environment then we should also take into account the proper voice characteristics, expressions and time length of voice announcements or interaction. Especially, the robotic voice should exhibit “empathy” towards the needs of the “green habitant”. Consequently, this scheme has the purpose of redefining the proper communication mechanisms that should be applied on already developed voice hardware which are intended for smart green residences. This scheme is compliant with an already developed protocol named as Reduced Ecological Footprints of Modern Facilities REFF [1].

**Materials and methods:** The primary purpose of this study is to place the proper foundations for designing a scheme which will include step by step information for constructing a voice protocol. This protocol includes 10 rules to be applied, even in already constructed systems. The first rule is the ability of the system to self-adapt in the presence of a human. This self-adaptation needs the implementation of micro-cameras along with microphones. The first rules’ function will be to monitor continuously the human habitant (in the field of vision of each device). This will help each Digital Processing System (incorporated in every system) or a general monitoring system to quick adapt to the subject’s emotional status. This will include the proper adaptation of robotic speech and of the optional emoticons’ projection on the screen of the smart device (e.g. refrigerator, washing machine, etc.). The 2nd rule is the adaptation of the robotic voice pitch. E.g. higher pitch would be more emotional. If a bad habitant’s condition is sensed, then the pitch (0–100) of the robot should be lower in order not to trigger further emotional burden. In this occasion, the voice will be perceived as less pleasant but will empower the habitant through its stronger perceived personality. Normal pitch has a mean value of 50, while the lower pitch exhibits a flat and monotonically observed sound [2]. The 3rd rule takes into consideration other variables such as volume, timbre and speech rate which their proper adjustment will lead to a more empathetic or non-empathetic robotic voice. The 4th rule will include the proper selection of gender’s (female, male) or a child’s voice (the latter only in the case of addressing to young children). Especially the last case of the robotic child’s voice will help towards avoiding accidents and towards the proper learning process of hazards. The robotic child’s voice and the proper figure projections on a screen will act in a mimetic manner from the child’s aspect towards avoiding the danger (primary prevention) in the case of an imminent misuse of an electric appliance inside a Green Residence. The 5th rule is specialized into creating a more humorous character when other rules won’t have the expected impact. The recommended type of humor as declared in [2] is the “innocent humor” which is definitely inoffensive. This type of self-adaptation will be furthermore empathetic and at the same time will try to pass in the mind of the habitant the key points towards avoiding dangers. In turn, this robotic behavior could help also the better social interaction of the habitant as it will improve his/hers mood. The 6th rule incorporates the feedback function which will be a common feature in all smart devices. The feedback function will transport in high data rates the information relevant to the mood of the habitant and other vital information to the next near device. Consequently, all devices will cooperate for the best possible safety even with the use of voice scenarios cooperation. This will be consisted of various types of voices when the system perceives high imminent danger. Network support should also be enabled in order to alert public health services of primary health care. The 7th rule contains update capabilities in order for the smart devices to be promptly reinforced with up-to-date voice functions. The 8th rule contains identification of voice disorder protocols. This function also includes conducting in parallel a minor psychological evaluation for prompting the habitant with proper counselling towards addressing to primary health care services. The 9th rule will include an advanced disguised protocol which will use Voice Handicap Index (VHI) for further obtaining the screening status of the habitants. The domains of VHI consist of the physical, functional and emotional parameters [3–7]. The 10th rule consists of quick logical decisions in order to identify and act rapidly in times of situations beyond danger (E.g. Sudden events such as quick escalated fire). The voice then becomes monotonic with low pitch, high volume and mean speech rate while system uses all available devices to quickly help the habitant to abandon the residence or green facility through voice commands.

**Results:** Fifty people (25 men and 25 women) constituting a typical group of subjects without any kind of clinical disorders will be recruited in order to participate in all 10-different kind of scenarios (character variations) inside a building complying to REFF Protocol [1]. Consequently, 10 independent groups will be formed which will participate only in one different scenario. This will certify that the answers in an impromptu questionnaire will be independent of comparison. The employed statistical strategies will include the correlation of same and different parameters inside the 10 different experiments. Also, Wilcoxon signed rank test in order to detect differences in ratings between the ten different scenarios. Moreover, Kruskal–Wallis tests will be applied being the most appropriate for such small samples. Also, if applicable, the Mann–Whitney U tests will compare the different “character” groups to a same data type collection. It is expected that people when annoyed will react efficiently in the presence of empathetic voice. Moreover, females will react differently to female empathetic voices as in [2]. This probably will show that female participants want more professionalism or probably a male voice instead of a female.

**Conclusions:** This research is a part of the construction of an enlarged protocol which will be generalized for all green buildings. Specifically, the “voice interaction” is a significant part of the Green environment as it humanizes the green technological environment offering more safety and better correspondence even to various types of habitants [8–9]. Also, an important factor is utilized which is the application of Primary Health Care. This idea also could be applied to “small talking places” in the dimension of ATMs inside Hospitals, Schools and Public Services.

**References**

1. Chronopoulos S, Kosma E, Tafiadis D et al. Reduced Ecological Footprints of Modern Facilities Introducing the Implementation of Advanced Wireless Technologies, and Human Resources’ Benefits. Communications and Network. 2018;10(01):11–29. 10.4236/cn.2018.101002
2. Niculescu A, van Dijk B, Nijholt A, Li H, See S. Making Social Robots More Attractive: The Effects of Voice Pitch, Humor and Empathy. International Journal of Social Robotics. 2013;5(2):171–191. 10.1007/s12369-012-0171-x
3. Tafiadis D, Chronopoulos S K, Kosma E I et al. Using Receiver Operating Characteristic Curve to Define the Cutoff Points of Voice Handicap Index Applied to Young Adult Male Smokers. Journal of Voice. 2018;32(4):443–448. 10.1016/j.jvoice.2017.06.007 
4. Tafiadis D, Kosma E I, Chronopoulos S K et al. Voice Handicap Index and Interpretation of the Cutoff Points Using Receiver Operating Characteristic Curve as Screening for Young Adult Female Smokers. Journal of Voice. 2018;32(1):64–69. 10.1016/j.jvoice.2017.03.009
5. Tafiadis D, Chronopoulos S, Siafaka V et al. Comparison of Voice Handicap Index Scores Between Female Students of Speech Therapy and Other Health Professions. Journal of Voice. 2017;31(5):583–588. 10.1016/j.jvoice.2017.01.013
6. Tafiadis D, Helidoni M, Chronopoulos S et al. Preliminary Receiver Operating Characteristic Analysis on Voice Handicap Index of Laryngeal Inflammation in Greek Patients. International Journal of Otolaryngology and Head & Neck Surgery. 2018;07(03):115–131. 10.4236/ijohns.2018.73014
7. Tafiadis D, Kosma E, Chronopoulos S, Voniati L, Ziavra N. A Preliminary Receiver Operating Characteristic Analysis on Voice Handicap Index Results of the Greek Voice-Disordered Patients. International Journal of Otolaryngology and Head & Neck Surgery. 2018;07(03):98–114. 10.4236/ijohns.2018.73013
8. Voniati L. Christopoulou M. Developing an Augmentative and Alternative Communication System for a Child with Autism Spectrum Disorder: A Case Study, American Journal of Health Research. 2017; 1(2): 58–62. 10.11648/j.ijcsd.20170102.15
9. Voniati L. Charalambous, I. Rett syndrome: Clinical recognition, communication skills and therapeutic intervention, Archives of Hellenic Medicine. 2018; 35(2): 188–197.

**Ethics Approval:** The study was approved by the Ethical Committee of Department of Speech Language Therapy (School of Health and Welfare Professions) TEI of Epirus

**Consent to publish:** Informed consent to publish has been obtained from each participant.

## A14 Revolutionizing e-learning with the proposition of Matrix Based-Artificial Intelligent Decisions (MB-AID) taking into account the best practices towards each person’s psychological condition

### Spyridon K. Chronopoulos^1,2^, Evangelia I. Kosma^3,4^, Dionysios Tafiadis^5^, Anastasios Skrivanos^6^, Ngoc-Tu Nguyen^7^, Kostas P Peppas^6^, Pantelis Angelidis^2^, Panos Kostarakis^1^

#### ^1^Physics Department, Electronics-Telecommunications and Applications Laboratory, University of Ioannina, Ioannina, Greece; ^2^Department of Informatics and Telecommunications Engineering, University of Western Macedonia, Kozani, Greece; ^3^Faculty of Medicine, School of Health Sciences, University of Thessaly, Biopolis, Larissa, Greece; ^4^Psychologist, Private Practice, Ioannina, Greece; ^5^Department of Speech & Language Therapy, Technological Educational Institute of Epirus, Ioannina, Greece; ^6^Department of Informatics and Telecommunications, University of Peloponnese, Akadimaikou G. K. Vlahou, Tripoli, Greece; ^7^Department of Computer Science & Engineering, University of Minnesota, Twin Cities, Minneapolis, MN 55455, USA

##### Spyridon K. Chronopoulos - schronopoulos@uowm.gr; spychro@gmail.com

*Annals of General Psychiatry* 2018, **17(Suppl 1):**A14

**Background:** Throughout the history of e-learning, the people with disabilities had to face a lot of omissions relevant to every aspect of their learning procedure [1]. These problems ranged from improper web designed pages to the improper way of confronting these types of our fellow citizens. This research takes into consideration the need for radical changes into e-learning process and especially the need for breakthrough changes towards e-class design while it introduces a new way for assessing people and especially patients suffering from severe psychological problems. The latter could be accomplished by properly calibrating the innovative e-class environment (by an Artificial Intelligent System) through audiovisual and self-perceived measurements [2]. These measurements could be split into various matrix-based robotic decisions for properly estimating the condition of a person interacting with the e-class environment. This in turn will not only benefit the final user from the aspect of best e-learning but also to properly warn him/her for potential psychological problems that could be addressed with the use of a clinician (E.g. Ophthalmologist, Psychiatrist, Psychologist, Speech Language Therapist, etc.). Also, the proposed system could be part of an advanced scheme such as the Reduced Ecological Footprints of Modern Facilities REFF [3].

**Materials and methods:** The research is focused on a protocol which introduces the way of designing and constructing a viable smart e-learning environment. Moreover, this protocol is so flexible that it can be implemented in a large variety of applications and systems. It consists of two parts. The first part describes the needed hardware along with the imminent rules of designing the proposed e-learning system. The second part is relevant to the proper mathematical conditions in order the system to behave autonomously. Specifically, the first part has two protocols (P1 and P2). The P1 dictates the use of a micro system including seamless video and audio capturing. The specifications include a very high resolution micro-camera and an audio system with sophisticated filtering capable of distinguishing several voice streams among a complicated audio sample. The P2 is relevant to the Digital Processing System (DSP) which recognizes every body movement and expression in order to analyze primarily the psychological and even the clinical condition if the latter is possible. The e-learning system exhibits a self-guided behavior. It auto arranges based on the type of the trainee’s character, disability or even his/hers psychological condition. Typical population will confront a screen split into two sides. In one side the video lecture of the trainer will be shown while in the other side various digital notes will be displayed. The system will react to the psychological condition of the trainee and it will sometimes focuses to one of the two displayed sides-parts. Nevertheless, if the trainees are individuals with disabilities then the system environment will radically change. E.g. A deaf person will see a screen with the trainer communicating in sign language while notes will be displayed with enlarged fonts. The procedure will be conducted automatically by the system based on its sensors. During also the whole training procedure various popup messages will interact with the trainee using artificial intelligence logic. The second part is relevant to the mechanism of the system’s decisions. This mechanism includes matrices which have at least four elements which are named as C1 (“advantageous decision”), C2 (“others’ effect”), C3 (“disadvantageous decision”), and C4 (“psychological value”). All their values are expressed in 10-points scale. C1 corresponds to a soft decision (0–10) relevant to the right-taken decision, C2 and C4 correspond again to a soft decision (ranging from − 10 to 10). C2 and C4 correspond to the user’s contribution to the final system’s decision and to the psychological condition. E.g. If C2 < 0 then the person has a negative psychological condition and vice versa. If C4 < 0 then the person has a strong effect to the system while else, the person has a weak effect. Finally, the C3 factor takes always positive values and it corresponds to the false-taken decision. With the proper algorithmic application of the aforementioned factors, the e-learning system will adapt while it could determine one’s mood or psychological condition.

**Results:** A small sample consisted of 60 people (30 men and 30 women) and a control group of 20 people (10 men and 10 women) will be tested in using the proposed system. Prior to testing procedure, the participants will be asked to fill in the SCL-90-R questionnaire in order to be determined their current psychological condition. After the procedure, the participants will be asked again to fill in two questionnaires. These two impromptu questionnaires will examine firstly the satisfaction from using the proposed system (questionnaire 1) and secondly the distant learner’s satisfaction (questionnaire 2) [4]. The statistical evaluation methods will include various tests such as Wilcoxon signed rank test in order to define the differences between various detected groups of psychological conditions and Mann–Whitney U for finding the differences of all groups compared to the control group. Also, depending on the type of distribution, other tests will be conducted such as t-test, Anova and cluster analysis [5–6]. Generally, a predicted result would be the higher efficiency in working with such an e-learning system along with revitalizing the will to study more.

**Conclusions:** The proposed research is multidimensional as it has 3 goals to accomplish. The first goal is the appropriate protocols to be applied in order the system to be consisted of the needed hardware. Then the system should be well designed in order the e-learning system to be self-adaptable to various different trainees’ needs [7–8]. The final goal will acquire significant results from an add-on research through the construction and validation of impromptu questionnaires. Consequently, this research will not only be beneficial from the aspect of proposing an innovative system but furthermore will involve the construction of two very significant questionnaires which will be updated to provide satisfaction evaluation of edge e-learning systems. Future benefits will also involve security recognition systems for detecting probable human threats for nearby habitants or workers.

**References**

1. Kinash S, Crichton S, Kim-Rupnow W. A Review of 2000–2003 Literature at the Intersection of Online Learning and Disability. American Journal of Distance Education. 2004;18(1):5–19. 10.1207/s15389286ajde1801_2
2. Tafiadis D, Chronopoulos S K, Kosma E I et al. Using Receiver Operating Characteristic Curve to Define the Cutoff Points of Voice Handicap Index Applied to Young Adult Male Smokers. Journal of Voice. 2018;32(4):443–448. 10.1016/j.jvoice.2017.06.007 
3. Chronopoulos S, Kosma E, Tafiadis D et al. Reduced Ecological Footprints of Modern Facilities Introducing the Implementation of Advanced Wireless Technologies, and Human Resources’ Benefits. Communications and Network. 2018;10(01):11–29. 10.4236/cn.2018.101002
4. Gameel B. Learner Satisfaction with Massive Open Online Courses. American Journal of Distance Education. 2017;31(2):98–111. 10.1080/08923647.2017.1300462
5. Tafiadis D, Kosma E I, Chronopoulos S K et al. Voice Handicap Index and Interpretation of the Cutoff Points Using Receiver Operating Characteristic Curve as Screening for Young Adult Female Smokers. Journal of Voice. 2018;32(1):64–69. 10.1016/j.jvoice.2017.03.009 
6. Tafiadis D, Chronopoulos S, Siafaka V et al. Comparison of Voice Handicap Index Scores Between Female Students of Speech Therapy and Other Health Professions. Journal of Voice. 2017;31(5):583–588. 10.1016/j.jvoice.2017.01.013
7. Voniati L. Christopoulou M. Developing an Augmentative and Alternative Communication System for a Child with Autism Spectrum Disorder: A Case Study, American Journal of Health Research. 2017; 1(2): 58–62. 10.11648/j.ijcsd.20170102.15
8. Voniati L. Charalambous, I. Rett syndrome: Clinical recognition, communication skills and therapeutic intervention, Archives of Hellenic Medicine. 2018; 35(2): 188–197.

**Ethics Approval:** The study was approved by the Ethical Committee of Department of Speech Language Therapy (School of Health and Welfare Professions) TEI of Epirus

**Consent to publish:** Informed consent to publish has been obtained from each participant.

## A15 Standalone Empathetic Intelligent Monitoring System (SEIMoS)

### Anastasios G. Skrivanos^1^, Evangelia I. Kosma^2,3^, Spyridon K. Chronopoulos^4,5^, Dionysios Tafiadis^6^, Kostas P. Peppas^1^, Ngoc-Tu Nguyen^7^, Periklis Papadopoulos^8^, Pantelis Angelidis^2^, Panos Kostarakis^4^

#### ^1^Department of Informatics and Telecommunications, University of Peloponnese, Akadimaikou G. K. Vlahou, Tripoli, Greece; ^2^Faculty of Medicine, School of Health Sciences, University of Thessaly, Biopolis, Larissa, Greece; ^3^Psychologist, Private Practice, Ioannina, Greece; ^4^Physics Department, Electronics-Telecommunications and Applications Laboratory, University of Ioannina, Ioannina, Greece; ^5^Department of Informatics and Telecommunications Engineering, University of Western Macedonia, Kozani, Greece; ^6^Department of Speech & Language Therapy, Technological Educational Institute of Epirus, Ioannina, Greece; ^7^Department of Computer Science & Engineering, University of Minnesota, Twin Cities, Minneapolis, MN 55455, USA; ^8^Physics Department, University of Ioannina, Ioannina, Greece

##### **Correspondence:** Spyridon K. Chronopoulos - schronopoulos@uowm.gr; spychro@gmail.com

*Annals of General Psychiatry* 2018, **17(Suppl 1):**A15

**Background:** In the modern era of vast communication and information dissemination the need for radical security measures must be addressed. Specifically, as the Internet has a tremendous influence on people and especially on younger ones, new threats are added to existing ones such as the Blue Whale phenomenon [1]. The latter is continuously growing with the corresponding suicide rate to equally arise while being already beyond acceptable values. Especially in some countries the percentage is really high denoting that immediate confrontation is needed. Many solutions can be found but the most proper should be found in any means of electronic monitoring and intervention. In turn, this autonomous monitoring system will exhibit empathetic characteristics. This system will have the capability of working locally but can also be connected to every network in order to alert security services for quick on-site intervention. The hardware part of the system will contain wired and wireless sensor nodes which will conduct measurements from various points of the body, as well as from various peripheral devices that are close to it. The software part will include a database and a local application to store the information acquired by sensors. In turn, the feedback will be processed algorithmically based on significant principals of psychotherapy in order the system to influence the users through multimedia interaction (empathetic approach) for the sake of their mental health. Also, additional features will be available for the sake of their relaxation or their stimulation for carrying on their work. The proposed scheme is named as Standalone Empathetic Intelligent Monitoring System (SEIMoS) which is based on Freudian psychoanalysis.

**Materials and methods:** This research has the purpose of investigating a viable solution towards the continuous clinical monitoring of people in order to act as precaution beneficial measure. By properly classifying the various stage procedures of SEIMoS such as its input information acquisition (by sensors) and the signal processing part, the system could be split into three major subsystems. The first subsystem includes all the sensory components, the second subsystem is directly related to the software innovative operation while the third subsystem is the algorithmic implementation of Freudian psychoanalysis into the software of the system. The latter enhances the system for exhibiting empathetic reactions in order to confront potential hazards such as imminent users’ suicide attempts. A small but significant detail is that each subsystem has digital signal processors (DSPs) acting as an additional intermediate security firewall against network attacks. Albeit this adds more complexity to the system, it is a needed part for not compromising the health of the monitored subjects in any case of potential security violation. The first subsystem includes various sensors, located or integrated in the keyboard, mouse, monitor screen, desk, footrest, inside buttons, chair, and in the perimeter of the user’s position. Additional supported sensors could be based on the body wireless sensors network (BWSN) technology [2]. Generally, these sensors will measure transpiration, pressure (diastolic and systolic), heartbeat, body temperature, and vibrations. Also, an integrated oximeter on a device such as a “mouse” could even provide other vital information. Furthermore, additional technologies will be part of this subsystem such as face recognition with high detail sensing for recognizing facial features like the movement of the eye’s pupil (expansion or contraction), of the eyebrows or even lips’ micro movements. Also, audio will be processed automatically regarding the spoken words and the voice quality relevant to potential disorders [3]. The second subsystem is the software system with the capability of combining different results for activating various procedures. One of the procedures could be the immediate self-adaptation of the system relevant to an imminent hazard. Then, the system could interact with the users in order to notify them or to “persuade” them to act differently. For example, if the users interact with “Blue Whale”, then the system will adapt and start to show messages or advertisements with subconscious messages. These will help changing their mood and consequently to make them abort the “Blue Whale” procedure relevant to an imminent suicide. Also, the system could even control devices compliant to IoT (Internet of Things). E.g. If a sensor (mouse device) sends the information of increased moisture or transpiration, along with a probable rise of user’s circumferential temperature then the air conditioning will be activated for adjusting the microclimate. Finally, the third subsystem is the heart of the proposed scheme as it acts based on Freudian Psychoanalysis which will be implemented as the decision maker into second subsystem. This theory claims that the pleasure principle drives a person towards the instinctive seeking of pleasure or death. This seeking is combined to the avoidance of pain for satisfying the biological and psychological needs. So, the system will constantly measure the satisfaction of the users and their psychological condition in order to identify if they experience pain (psychological or physical). Consequently, the id (type of the personality) of a user could be easily understood by the system when six values (10-scale) will have been measured. These are the positive satisfaction (PS—seeking of pleasure), the negative satisfaction 1 (seeking of death—aggressive towards others), the negative satisfaction 2 (seeking of death—self aggressive), avoidance of pain 1 and 2 which exhibit positive or negative values respectively, and finally the estimation or the already known knowledge of clinical condition. Even if this condition is not known this is not a prohibitive factor for the system to conclude to an action towards the user. E.g. If a user exhibits positive satisfaction and at the same time a negative avoidance of pain then these measurements indicate a potential masochistic subject. Also, if a user is measured of having negative satisfaction and positive avoidance of pain then he is a not a potential victim of “Blue Whale”. If the aforementioned six factors are implemented inside the third subsystem then the latter can understand the users’ condition and accordingly to act empathetically for helping them to supersede various hazardous psychological or clinical emerged conditions.

**Results:** This research will be carried out in at least three different places which would be inside typical buildings, inside a LEED building [4] and finally a building which shares elements from the previous two types of buildings. 150 people (men and women) will be split into three teams of 50 subjects. All the subjects should be analyzed prior to the use of SCL-90-R in order to determine if they are typical users [5–7]. Also, another impromptu questionnaire will be filled in for determining the type of a possible physical illness and for correlating the results of this category of subjects to possible system’s unpredicted results. After the procedure, all the subjects will be evaluated again with a new impromptu questionnaire based on the system’s employed Freudian Psychoanalysis’ factors. Then, statistical analysis will be conducted along with the goal of finding correlations between typical people having physical illnesses and various confronted conditions such as “Blue Whale” phenomenon. The anticipated tests will probably include cluster analysis along with Wilcoxon signed rank tests. In fact, a prediction would definitely be that typical population, having a physical illness and being in a negative avoidance of pain, would be candidate of heavy influence not only through Internet but also in real life.

**Conclusions:** This study has two perspectives. One is the research towards creating an evolved interactive electronic system while the other prospective is to construct a new questionnaire which will be even used for monitoring purposes. Specifically, the Standalone Empathetic Intelligent Monitoring System aims to promote the decisions of every electronic interactive system to a higher intelligence level (with the support of sensory technology). The constant use will enhance this system as it will learn and adapt according to special conditions such as imminent suicides, deaths from other causes or even typical needs of a better micro climate inside a typical or a green house. The prototyping’s anonymous results will be saved to a database in order to discover new factors of estimating the clinical condition of a person along with the evaluation of the results from the system’s intervention to users’ decisions. Another significant result would be the validation of a new questionnaire which will be further enhanced based on the feedback from the electronic prototype platform. This questionnaire based on Freudian Psychoanalysis will be a significant predictive tool in the hands of a clinician.

**References**

1. Belfort E, Miller L. Relationship Between Adolescent Suicidality, Self-Injury, and Media Habits. Child Adolesc Psychiatr Clin N Am. 2018;27(2):159–169. 10.1016/j.chc.2017.11.004
2. Angelidis P. Personalised physical exercise regime for chronic patients through a wearable ICT platform. International Journal of Electronic Healthcare. 2010;5(4):355. 10.1504/ijeh.2010.036207
3. Tafiadis D, Chronopoulos S K, Kosma E I et al. Using Receiver Operating Characteristic Curve to Define the Cutoff Points of Voice Handicap Index Applied to Young Adult Male Smokers. Journal of Voice. 2018;32(4):443–448. 10.1016/j.jvoice.2017.06.007 
4. Chronopoulos S, Kosma E, Tafiadis D et al. Reduced Ecological Footprints of Modern Facilities Introducing the Implementation of Advanced Wireless Technologies, and Human Resources’ Benefits. Communications and Network. 2018;10(01):11–29. 10.4236/cn.2018.101002
5. Tafiadis D, Kosma E I, Chronopoulos S K et al. Voice Handicap Index and Interpretation of the Cutoff Points Using Receiver Operating Characteristic Curve as Screening for Young Adult Female Smokers. Journal of Voice. 2018;32(1):64–69. 10.1016/j.jvoice.2017.03.009 
6. Tafiadis D, Kosma E, Chronopoulos S et al. Acoustic and Perceived Measurements Certifying Tango as Voice Treatment Method. Journal of Voice. 2018;32(2):256.e13–256.e24. 10.1016/j.jvoice.2017.05.016
7. Tafiadis D, Chronopoulos S, Siafaka V et al. Comparison of Voice Handicap Index Scores Between Female Students of Speech Therapy and Other Health Professions. Journal of Voice. 2017;31(5):583–588. 10.1016/j.jvoice.2017.01.013

**Ethics Approval:** The study was approved by the Ethical Committee of Department of Speech Language Therapy (School of Health and Welfare Professions) TEI of Epirus

**Consent to publish:** Informed consent to publish has been obtained from each participant.

## A16 How robots could reach humanity with the implementation of Advanced Emotional Signalling in Algorithmically Structured Systems (AESASS)

### Spyridon K. Chronopoulos^1,2^, Evangelia I. Kosma^3,4^, Dionysios Tafiadis^5^, Anastasios G. Skrivanos^6^, Ngoc-Tu Nguyen^7^, Kostas P. Peppas^6^, Pantelis Angelidis^2^, Panos Kostarakis^1^

#### ^1^Physics Department, Electronics-Telecommunications and Applications Laboratory, University of Ioannina, Ioannina, Greece; ^2^Department of Informatics and Telecommunications Engineering, University of Western Macedonia, Kozani, Greece; ^3^Faculty of Medicine, School of Health Sciences, University of Thessaly, Biopolis, Larissa, Greece; ^4^Psychologist, Private Practice, Ioannina, Greece; ^5^Department of Speech & Language Therapy, Technological Educational Institute of Epirus, Ioannina, Greece; ^6^Department of Informatics and Telecommunications, University of Peloponnese, Akadimaikou G. K. Vlahou, Tripoli, Greece; ^7^Department of Computer Science & Engineering, University of Minnesota, Twin Cities, Minneapolis, MN 55455, USA

##### **Correspondence:** Spyridon K. Chronopoulos - schronopoulos@uowm.gr; spychro@gmail.com

*Annals of General Psychiatry* 2018, **17(Suppl 1):**A16

**Background:** Robots as well-known are complex machines constituted of artificial parts and digital logic structures. Two key points need special attention in order an artificial humanoid to be finely developed. Firstly, the artificial structure must resemble to a human not only in the figure but moreover in expressed movements accomplishing a body language very close to that of their owner while this artificial body should include sensors network (BWSN) technology [1]. Secondly, the artificial brain must exhibit seamless decisions and if needed quick responses taking into consideration an empathetic employed nature of behavior. The latter could be accomplished with the use of 5-stage iterative logic which guarantees the best possible result and a unique behavior even when compared between the same robot models. In this point of development all precautions must take into account that the artificial developed characters should conform to a significant standard. Their auto-development should not constitute an abuse towards their owner’s safety and generally towards every other human’s clinical condition. Additionally, a self-robot check is proposed based on SCL90-R questionnaire as soon as the artificial character will be fully-deployable.

**Materials and methods:** This work is centered on five primal principles-stages. Iterative logic is employed in order the system to self-adapt to the best solution. The latter is accomplished by using soft approximation through various stages and finally a feedback loop to constantly monitor new apprehended data versus past decisions. In this way, the behavior of the robot is constantly changing in order to adapt to their owner’s manners and behavior. The decision making is enhanced by using double precision 7-likert scale i.e. the grade of every estimation has two parts. One part is the 1–7 positive value relevant to what the owner wants while the other part (1–7 value) corresponds to how much empathetic behavior is exhibited by the robot. The later depends also to past conditions and it could take different values even when the first part has the same value. This means that the robot expresses their mood and consequently an artificial intelligence behavior which is accomplished by taking into consideration various conditions even the climate itself. The iterative empathetic behavior is consisted of five stages. The first stage is a comparator of the total score of current and past condition while the second is the estimation of the optical data. Then a new soft decision (third stage) uses the newly estimated optical data in conjunction to the audio evaluation of the environment. This denotes the necessity of always taking into consideration the environmental conditions along with the data from audiovisual sensing. The fourth part estimates the value of pressure in various body parts of the robot (3D perception of the environment). E.g. when a robot senses a lot of pressure (hit) while talking to a subject then this is estimated as a value of the anger towards their structure. The fifth part is more complicated as it uses a version of an impromptu questionnaire based on SCL-90-R clinical evaluation not only for the human but also for the structure itself. Then a feedback loop passes through a memory block which has stored the significant past conditions for comparing them to the current one (consisted of five likert scores) and in this way to produce a final Likert score (five parts) which then is guided back to the first stage. The output of the final decision is each time after fifth stage. Especially the memory block is very important as it utilizes the humans’ logic of metamemory (a type of metacognition also known as Socratic awareness) in order to self-perceive and be guided by past knowledge and decisions. This is essential for developing a true robotic ability of Empathy.

**Results:** This research involves a total sample of 30 people (15 men and 15 women) in order to be conducted a preliminary test of reaction towards Advanced Emotional Signaling in Algorithmically Structured System (AESASS) inside typical and LEED buildings [2]. The latter is in fact a true empathetic robot exhibiting a self-acquired and not conventional or programmed behavior compared to other robotic structures. This behavior will be analyzed and tested when it is experienced by 30 people. Before the testing procedure, each subject will fill the SCL-90-R questionnaire for verifying that their psychological condition is among typical population. Also, the subjects should not exhibit any disorder even those related to voice [3, 4]. Also, Active—Empathetic Listening Scale (AELS) along with Toronto Empathy questionnaire will be used in this research in order to determine the empathetic level of each subject. After the robotic experience a new impromptu questionnaire will be administered for estimating the grade of satisfaction from the interaction with the robot. The evaluation will be conducted by using (if possible) ANNOVA between various unrelated features in order to determine how these could be related to the robot’s feedback experience. Also, skewed variables will be checked and if possible Wilcoxon signed test along with Mann–Whitney U tests will be applied to the data set. Especially Kruskal–Wallis tests will be conducted as they are one of the best statistical procedures for manipulating small samples. Then cluster analysis should exhibit important results. This will help grouping objects for better analyzing the behavior and estimation of the subjects relevant to the robotic experience.

**Conclusions:** This work involves the construction and estimation of a robotic structure which will employ advanced empathetic logic and self-awareness with the use of an internal robotic SCL-90-R version. This version will help towards the proper self-analysis of the robot and thus will give the capability of its application to other robotic systems. Moreover, the new impromptu satisfaction questionnaire will be an important new estimator of the robotic behavior of various systems and structures. It is expected that the subjects will be keen to a robotic structure but they will be very susceptible to a structure exhibiting Empathy and not an artificial Behavior. Other future benefits will involve the recording of this satisfaction in conjunction to time, while robotic technology will keep on moving forward. If the scores increase radically from a time point and then, this will declare an even higher robotic evolution.

**References**

1. Angelidis P. Personalised physical exercise regime for chronic patients through a wearable ICT platform. International Journal of Electronic Healthcare. 2010;5(4):355. 10.1504/ijeh.2010.036207
2. Chronopoulos S, Kosma E, Tafiadis D et al. Reduced Ecological Footprints of Modern Facilities Introducing the Implementation of Advanced Wireless Technologies, and Human Resources’ Benefits. Communications and Network. 2018;10(01):11–29. 10.4236/cn.2018.101002
3. Tafiadis D, Chronopoulos S K, Kosma E I et al. Using Receiver Operating Characteristic Curve to Define the Cutoff Points of Voice Handicap Index Applied to Young Adult Male Smokers. Journal of Voice. 2018;32(4):443–448. 10.1016/j.jvoice.2017.06.007  
4. Tafiadis D, Kosma E I, Chronopoulos S K et al. Voice Handicap Index and Interpretation of the Cutoff Points Using Receiver Operating Characteristic Curve as Screening for Young Adult Female Smokers. Journal of Voice. 2018;32(1):64–69. 10.1016/j.jvoice.2017.03.009 

**Ethics Approval:** The study was approved by the Ethical Committee of Department of Speech Language Therapy (School of Health and Welfare Professions) TEI of Epirus

**Consent to publish:** Informed consent to publish has been obtained from each participant.

## A17 Estimation through data analysis of the self-perceived levels relevant to the reading abilities of typical Greek female adults: evidence based data using adults reading questionnaire

### Anna Alexandropoulou1, Evangelia I. Kosma2,3, Louiza Voniati^4^, Spyridon K. Chronopoulos^5,6^, Nikoleta Alexopoulou^7^, Dionysios Tafiadis^1^

#### ^1^Department of Speech & Language Therapy, Technological Educational Institute of Epirus, Ioannina, Greece; ^2^Faculty of Medicine, School of Health Sciences, University of Thessaly, Biopolis, Larissa, Greece; ^3^Psychologist, Private Practice, Mihail Aggelou 18, Ioannina, Greece; ^4^Department of Speech & Language Therapy, European University of Cyprus, Nicosia, Cyprus; ^5^Department of Informatics and Telecommunications Engineering, University of Western Macedonia, Kozani, Greece; ^6^Department of Computer Engineering, Technological Educational Institute of Epirus, Arta, Greece; ^7^Department of Humanities and Social Sciences, University of Strathclyde, Glasgow, United Kingdom

##### **Correspondence:** Dionysios Tafiadis - d.tafiadis@ioa.teiep.gr; tafiadis@gmail.com

*Annals of General Psychiatry* 2018, **17(Suppl 1):**A17

**Background:** Reading abilities in women seem to alter as age progresses and as cultural framework changes. Estrogen related changes also appear to contribute in reading abilities. Moreover, research shows that analphabetism affects women more than men which results in literacy difficulties. However, it is important to determine how different ages perceive their reading abilities as such data can prove useful to clinicians. In this study, we attempted to create evidenced based data for the self-perceived levels of reading abilities for typical Greek women using Adults Reading Questionnaire.

**Materials and methods:** 308 typical female literate adults aged from 18 to 85 years old (Μ = 44.25, SD = 13.526) participated in this study. The sample’s education varied in years. The validated Greek version of ARQ questionnaire was administered in order to self-evaluate their reading abilities.

**Results:** The study showed statistical significant discrepancies between age groups. Significant differences were reported for ARQ total score [H95) = 15.745, p < 0.050] as well for specific questions [question 1–question 5, question 10–question 11 and question14 (p < 0.050)]. The worst scoring was observed for the age group of 70 plus.

**Conclusions:** The results exposed significant contributes of the age factor in the formation of the scores. As expected, older individuals scored higher that younger subjects in most of the questions as in other questionnaires [1–8]. From this, we can conclude that women of older ages face greater difficulties during reading, maybe due to natural aging or undetected disorders.

**References**

1. Tafiadis D, Chronopoulos S K, Kosma E I et al. Using Receiver Operating Characteristic Curve to Define the Cutoff Points of Voice Handicap Index Applied to Young Adult Male Smokers. Journal of Voice. 2018;32(4):443–448. 10.1016/j.jvoice.2017.06.007
2. Tafiadis D, Kosma E I, Chronopoulos S K et al. Voice Handicap Index and Interpretation of the Cutoff Points Using Receiver Operating Characteristic Curve as Screening for Young Adult Female Smokers. Journal of Voice. 2018;32(1):64–69. 10.1016/j.jvoice.2017.03.009
3. Tafiadis D, Chronopoulos S, Siafaka V et al. Comparison of Voice Handicap Index Scores Between Female Students of Speech Therapy and Other Health Professions. Journal of Voice. 2017;31(5):583–588. 10.1016/j.jvoice.2017.01.013
4. Tafiadis D, Kosma E, Chronopoulos S et al. Acoustic and Perceived Measurements Certifying Tango as Voice Treatment Method. Journal of Voice. 2018;32(2): 256.e13–256.e24. 10.1016/j.jvoice.2017.05.016
5. Voniati L. School Based Speech and Language Therapists’ Perceptions of MLU-w and Its Use. Science Journal of Education. 2015; 3(6):119–125. 10.11648/j.sjedu.20150306.11
6. Voniati L. Mean Length of Utterance in CYG-speaking children. Journal of Greek Linguistics. 2016; 16(1): 117–140.
7. Voniati L. Christopoulou M. Developing an Augmentative and Alternative Communication System for a Child with Autism Spectrum Disorder: A Case Study, American Journal of Health Research. 2017; 1(2): 58–62. 10.11648/j.ijcsd.20170102.15
8. Voniati L. Charalambous, I. Rett syndrome: Clinical recognition, communication skills and therapeutic intervention, Archives of Hellenic Medicine. 2018; 35(2): 188–197.

**Ethics Approval:** The study was approved by the Ethical Committee of Department of Speech Language Therapy (School of Health and Welfare Professions) TEI of Epirus.

**Consent to publish:** Informed consent to publish has been obtained from each participant.

## A18 Estimation through data analysis of the self-perceived levels relevant to the reading abilities of typical Greek male adults: evidence based data using adults reading questionnaire

### Anna Alexandropoulou^1^, Evangelia I. Kosma^2,3^, Louiza Voniati^4^, Spyridon K. Chronopoulos^5,6^, Dionysios Tafiadis^1^

#### ^1^Department of Speech & Language Therapy, Technological Educational Institute of Epirus, Ioannina, Greece; ^2^Faculty of Medicine, School of Health Sciences, University of Thessaly, Biopolis, Larissa, Greece; ^3^Psychologist, Private Practice, Mihail Aggelou 18, Ioannina, Greece; ^4^Department of Speech & Language Therapy, European University of Cyprus, Nicosia, Cyprus; ^5^Department of Informatics and Telecommunications Engineering, University of Western Macedonia, Kozani, Greece; ^6^Department of Computer Engineering, Technological Educational Institute of Epirus, Arta, Greece

##### **Correspondence:** Dionysios Tafiadis - d.tafiadis@ioa.teiep.gr; tafiadis@gmail.com

*Annals of General Psychiatry* 2018, **17(Suppl 1):**A18

**Background:** Many problems arise for adults with literacy difficulties in the daily demanding environment. Those problems are probably the outcome of undetected and untreated literacy symptoms (as reading disabilities) at childhood. Recent findings suggest that such difficulties are more common amongst men than women. Considering the above, the purpose of this study was to record how typical male adults perceived their reading abilities using Adults Reading Questionnaire in order to create evidence-based data for their population in Greece.

**Materials and methods:** The validated Greek version of Adult Reading Questionnaire was used for this study. It was administered to 310 typical literate Greek men of 18 to 84 years of age (Μ = 44.49, SD = 14.174). The sample was gathered form many regions of Greece while its years of educations varied.

**Results:** Statistical analysis pointed out significant differences between all age subgroups for ARQ’s total score [H(5) = 15.4382, p < 0.005]. Particularly, statistical significant differences were uncovered in 8 out of 39 questions of ARQ checklist. Higher negative scores observed for the age group of over 70 years and the rest subgroups.

**Conclusions:** The effect of age might not be a significant parameter to be taken into account during self-assessment of speech abilities for typical females. Overall, the awareness of co-ordination difficulties seems to be present mostly as age progresses as in other researches [1–8], but more research is needed for different type of populations such as those of pathological type.

**References**

1. Tafiadis D, Chronopoulos S K, Kosma E I et al. Using Receiver Operating Characteristic Curve to Define the Cutoff Points of Voice Handicap Index Applied to Young Adult Male Smokers. Journal of Voice. 2018;32(4):443–448. 10.1016/j.jvoice.2017.06.007
2. Tafiadis D, Kosma E I, Chronopoulos S K et al. Voice Handicap Index and Interpretation of the Cutoff Points Using Receiver Operating Characteristic Curve as Screening for Young Adult Female Smokers. Journal of Voice. 2018;32(1):64–69. 10.1016/j.jvoice.2017.03.009
3. Tafiadis D, Chronopoulos S, Siafaka V et al. Comparison of Voice Handicap Index Scores Between Female Students of Speech Therapy and Other Health Professions. Journal of Voice. 2017;31(5):583–588. 10.1016/j.jvoice.2017.01.013
4. Tafiadis D, Kosma E, Chronopoulos S et al. Acoustic and Perceived Measurements Certifying Tango as Voice Treatment Method. Journal of Voice. 2018;32(2): 256.e13–256.e24. 10.1016/j.jvoice.2017.05.016
5. Voniati L. School Based Speech and Language Therapists’ Perceptions of MLU-w and Its Use. Science Journal of Education. 2015; 3(6):119–125. 10.11648/j.sjedu.20150306.11
6. Voniati L. Mean Length of Utterance in CYG-speaking children. Journal of Greek Linguistics. 2016; 16(1): 117–140.
7. Voniati L. Christopoulou M. Developing an Augmentative and Alternative Communication System for a Child with Autism Spectrum Disorder: A Case Study, American Journal of Health Research. 2017; 1(2): 58–62. 10.11648/j.ijcsd.20170102.15
8. Voniati L. Charalambous, I. Rett syndrome: Clinical recognition, communication skills and therapeutic intervention, Archives of Hellenic Medicine. 2018; 35(2): 188–197.

**Ethics Approval:** The study was approved by the Ethical Committee of Department of Speech Language Therapy (School of Health and Welfare Professions) TEI of Epirus.

**Consent to publish:** Informed consent to publish has been obtained from each participant.

## A19 Using data analysis for the estimation of the self-perceived levels of speech coordination for typical Greek female adults: evidence based data for adult developmental co-ordination disorders/dyspraxia checklist

### Anna Alexandropoulou^1^, Evangelia I. Kosma^2,3^, Louiza Voniati^4^, Spyridon K. Chronopoulos^5,6^, Dionysios Tafiadis^1^

#### ^1^Department of Speech & Language Therapy, Technological Educational Institute of Epirus, Ioannina, Greece; ^2^Faculty of Medicine, School of Health Sciences, University of Thessaly, Biopolis, Larissa, Greece; ^3^Psychologist, Private Practice, Mihail Aggelou 18, Ioannina, Greece; ^4^Department of Speech & Language Therapy, European University of Cyprus, Nicosia, Cyprus; ^5^Department of Informatics and Telecommunications Engineering, University of Western Macedonia, Kozani, Greece; ^6^Department of Computer Engineering, Technological Educational Institute of Epirus, Arta, Greece

##### **Correspondence:** Dionysios Tafiadis - d.tafiadis@ioa.teiep.gr; tafiadis@gmail.com

*Annals of General Psychiatry* 2018, **17(Suppl 1):**A19

**Background:** Coordination of speech musculature is strongly associated with neurological dis- orders also resulting from natural aging. Whichever is the cause, an important part of the assessment is the use of self-reported tools [1–8] to determine the patient’s needs and requests. One is the Adult Developmental Co-ordination Disorders/Dyspraxia Checklist (ADC). Purpose of this study was to find how females perceived their co-ordination difficulties-abilities using ADC and how this perception differed through adult lifespan.

**Materials and methods:** The validated Greek version of ADC (a 39-items list) was used for this study. It was administered to 235 typical Greek females from 20 till 84 years of age (Μ = 49.43, SD = 17.800) to self-assess their motor functioning. The translation of the questionnaire was carried out in accordance with the minimum translation criteria.

**Results:** Statistical analysis revealed significant differences between all age subgroups for ARQ’s total score [H(5) = 22.438, p = 0.000]. Specifically, differences were found in question 1, question 3, question 5, question 6, question 10, question 11 and question13 (p < 0.050). The higher scoring was recorded from the eldest age group (70+) in contrast to the rest age groups.

**Conclusions:** In conclusion, age seems to be a significant parameter in order to be taken into account during self-assessment of reading abilities. Elder individuals scored higher compared to younger participants which this may be due to their lesser years of study rate than elderly Greek population. Consequently, more in-depth research must be conducted since studies showed that literacy difficulties share common causality with mental disorders and thus their timely evaluation is of great importance.

**References**

1. Tafiadis D, Chronopoulos S K, Kosma E I et al. Using Receiver Operating Characteristic Curve to Define the Cutoff Points of Voice Handicap Index Applied to Young Adult Male Smokers. Journal of Voice. 2018;32(4):443–448. 10.1016/j.jvoice.2017.06.007
2. Tafiadis D, Kosma E I, Chronopoulos S K et al. Voice Handicap Index and Interpretation of the Cutoff Points Using Receiver Operating Characteristic Curve as Screening for Young Adult Female Smokers. Journal of Voice. 2018;32(1):64–69. 10.1016/j.jvoice.2017.03.009
3. Tafiadis D, Chronopoulos S, Siafaka V et al. Comparison of Voice Handicap Index Scores Between Female Students of Speech Therapy and Other Health Professions. Journal of Voice. 2017;31(5):583–588. 10.1016/j.jvoice.2017.01.013
4. Tafiadis D, Kosma E, Chronopoulos S et al. Acoustic and Perceived Measurements Certifying Tango as Voice Treatment Method. Journal of Voice. 2018;32(2): 256.e13–256.e24. 10.1016/j.jvoice.2017.05.016
5. Voniati L. School Based Speech and Language Therapists’ Perceptions of MLU-w and Its Use. Science Journal of Education. 2015; 3(6):119–125. 10.11648/j.sjedu.20150306.11
6. Voniati L. Mean Length of Utterance in CYG-speaking children. Journal of Greek Linguistics. 2016; 16(1): 117–140.
7. Voniati L. Christopoulou M. Developing an Augmentative and Alternative Communication System for a Child with Autism Spectrum Disorder: A Case Study, American Journal of Health Research. 2017; 1(2): 58–62. 10.11648/j.ijcsd.20170102.15
8. Voniati L. Charalambous, I. Rett syndrome: Clinical recognition, communication skills and therapeutic intervention, Archives of Hellenic Medicine. 2018; 35(2): 188–197.

**Ethics Approval:** The study was approved by the Ethical Committee of Department of Speech Language Therapy (School of Health and Welfare Professions) TEI of Epirus.

**Consent to publish:** Informed consent to publish has been obtained from each participant.

## A20 Finding through statistical analysis the self-perceived levels of speech coordination for typical Greek male adults: evidence based data using adult developmental co-ordination disorders/dyspraxia checklist

### Anna Alexandropoulou^1^, Evangelia I. Kosma^2,3^, Louiza Voniati^4^, Spyridon K. Chronopoulos^5,6^, Dionysios Tafiadis^1^

#### ^1^Department of Speech & Language Therapy, Technological Educational Institute of Epirus, Ioannina, Greece; ^2^Faculty of Medicine, School of Health Sciences, University of Thessaly, Biopolis, Larissa, Greece; ^3^Psychologist, Private Practice, Mihail Aggelou 18, Ioannina, Greece; ^4^Department of Speech & Language Therapy, European University of Cyprus, Nicosia, Cyprus; ^5^Department of Informatics and Telecommunications Engineering, University of Western Macedonia, Kozani, Greece; ^6^Department of Computer Engineering, Technological Educational Institute of Epirus, Arta, Greece

##### **Correspondence:** Dionysios Tafiadis - d.tafiadis@ioa.teiep.gr; tafiadis@gmail.com

*Annals of General Psychiatry* 2018, **17(Suppl 1):**A20

**Background:** The motor that demands of speech production can be compromised by normal aging processes and thus it could change an individual’s precision and fluency. It is important to have individualized data on the motor difficulties which any person might face throughout his/her life to make the clinical process more efficient and client-oriented. To this direction self-reported questioner provide valuable information about clients’ perception for the clinical condition they experience, such as Adult Developmental Co-ordination Disorders/Dyspraxia Checklist (ADC) which is a 37-items Likert scale questionnaire. This study aimed to determine the self-perceived levels of motor abilities for typical adult males.

**Materials and methods:** The validated version of the ADC was administered in a total of 228 healthy adult men from 20 to 84 years of age (M = 49.43, SD = 17.800). Participants that had a history of neurological and mental illness or were illiterate were not included in this sample.

**Results:** The analysis showed significant differences between all age subgroups for the total score of ADC [H(5) = 12.060, p < 0.050]. Furthermore, statistical significant differences were observed even when the age subgroups compared as pairs. Also, significant differences were noticed for 14 out of 37 questions.

**Conclusions:** From the above it is determined that age is an important factor when it comes to co-ordination difficulties for male adults in an almost predictable way. As age progresses the perception of coordination of musculature probably becomes more of a challenge for the male population as in other researches [1–8].

**References**

1. Tafiadis D, Chronopoulos S K, Kosma E I et al. Using Receiver Operating Characteristic Curve to Define the Cutoff Points of Voice Handicap Index Applied to Young Adult Male Smokers. Journal of Voice. 2018;32(4):443–448. 10.1016/j.jvoice.2017.06.007
2. Tafiadis D, Kosma E I, Chronopoulos S K et al. Voice Handicap Index and Interpretation of the Cutoff Points Using Receiver Operating Characteristic Curve as Screening for Young Adult Female Smokers. Journal of Voice. 2018;32(1):64–69. 10.1016/j.jvoice.2017.03.009
3. Tafiadis D, Chronopoulos S, Siafaka V et al. Comparison of Voice Handicap Index Scores Between Female Students of Speech Therapy and Other Health Professions. Journal of Voice. 2017;31(5):583–588. 10.1016/j.jvoice.2017.01.013
4. Tafiadis D, Kosma E, Chronopoulos S et al. Acoustic and Perceived Measurements Certifying Tango as Voice Treatment Method. Journal of Voice. 2018;32(2): 256.e13–256.e24. 10.1016/j.jvoice.2017.05.016
5. Voniati L. School Based Speech and Language Therapists’ Perceptions of MLU-w and Its Use. Science Journal of Education. 2015; 3(6):119–125. 10.11648/j.sjedu.20150306.11
6. Voniati L. Mean Length of Utterance in CYG-speaking children. Journal of Greek Linguistics. 2016; 16(1): 117–140.
7. Voniati L. Christopoulou M. Developing an Augmentative and Alternative Communication System for a Child with Autism Spectrum Disorder: A Case Study, American Journal of Health Research. 2017; 1(2): 58–62. 10.11648/j.ijcsd.20170102.15
8. Voniati L. Charalambous, I. Rett syndrome: Clinical recognition, communication skills and therapeutic intervention, Archives of Hellenic Medicine. 2018; 35(2): 188–197.

**Ethics Approval:** The study was approved by the Ethical Committee of Department of Speech Language Therapy (School of Health and Welfare Professions) TEI of Epirus.

**Consent to publish:** Informed consent to publish has been obtained from each participant.

## A21 KMDRS and the assessment of peripartum depression

### Lavinia De Chiara^1^, Alexia Koukopoulos^2,3^, Giovanni Manfredi^1,3^, Gloria Angeletti^1,3^, Gabriele Sani^1,3^

#### ^1^NeSMOS Department, Sapienza University of Rome, Rome, Italy; ^2^Umberto I Hospital, Psychiatry Department, Rome, Italy; ^3^Centro Lucio Bini, Rome, Italy

##### **Correspondence:** Lavinia De Chiara - laviniadechiara@gmail.com

*Annals of General Psychiatry* 2018, **17(Suppl 1):**A21

**Background:** Most studies about mood disorders during the perinatal period have focused almost exclusively on typical depression [1]. Less attention has been paid to the mixed symptoms. There is a significant subpopulation of patients during pregnancy and postpartum period with depressive symptoms who has been classified as non-responder to first-line antidepressant treatments [2]. They get nervous, irritable, demanding, with psychic agitation and racing thoughts. When these symptoms are misdiagnosed, treatment will probably not be optimal for that woman with possible severe consequences. Many studies in the literature now confirm that the use of antidepressants in this patients could result in an emergence of manic or mixed symptoms and a higher risk of suicide [3]. The diagnostic evaluation of women with peripartum depression should include questions on mixed symptoms. Early recognition and clinical diagnosis of peripartum mixed depression is an important prerequisite to offer to these patients an adequate treatment and to prevent the negative impact on their children’s development.

The primary aim of this study was to detect mixed symptoms among peripartum mood episodes.

The secondary aim was to evaluate the performance of the KMDRS a new scale [4] in a sample of women during peripartum period.

**Materials and Methods**: The study includes 60 women with a new episode during pregnancy or at least within 12 months postpartum evaluated by the Center for Prevention and Treatment of Women’s Mental Health at Sant’Andrea Hospital of Rome. Patients were evaluated by The Edinburgh Postnatal Depression Scale (EPDS), the Hamilton Rating Scale For Anxiety (HAM-A), the Hamilton Rating Scale For Depression (HAM-D), the Young Mania Rating Scale (YMRS), the Brief Psychiatric Rating Scale (BPRS), the Clinical Global Impression (CGI) and The Koukopoulos Mixed Depression Rating Scale (KMDRS).

**Results**: Mixed symptoms were clinically evaluated as present in 20 patients (33.4%) whereas were absent in 40 patients. Women with mixed symptoms had significantly higher KMDRS mean scores (*p* = 0.005). BPRS mean scores were significantly higher in women with mixed symptoms than in women without mixed symptoms (*p* = 0.000). Women with mixed symptoms had almost significant higher CGI severity than women without mixed symptoms (*p* = 0.006).

Seven of the 14 items of the KMDRS had significant higher mean scores within patients with peripartum mixed symptoms compared with patients without peripartum mixed symptoms. Significant items were Emotional Lability (*p *= 0.040), Subjective Feelings of Irritability (*p *= 0.002), Overt Expression of Irritability (*p *= 0.001), Racing or Crowded Thoughts (*p *= 0.001), Inner Tension (*p *= 0.035), Suicidal Impulsiveness (*p *= 0.007) and Sexuality (*p *= 0.015)

**Limitations:** We use the “mixed symptoms” definition not according to DSM-5 mixed depression criteria, but according to the criteria for mixed depression proposed by Koukopoulos and collaborators [5]

**Conclusions**: The KMDRS was the only scale able to detect mixed symptoms. Women with mixed symptoms had higher CGI and BPRS scores which could mean that mixed depression was a more severe type of depression among our sample.

Most of the symptoms characterizing those women with peripartum mixed episodes would have been excluded by the DSM-5 definition.

We found the Koukopoulos Mixed Depression Rating Scale a very helpful tool among our peripartum patients to detect mixed depressions and better address management decisions.

**References**

1. Viguera AC, Tondo L, Koukopoulos AE, Reginaldi D, Lepri B, Baldessarini RJ. Episodes of mood disorders in 2,252 pregnancies and postpartum periods. Am J Psychiatry. 2011;168:1179–85.
2. Pope CJ, Sharma V, Sommerdyk C, Mazmanian D. Antidepressants and recurrence of depression in the postpartum period. Arch Womens Ment Health. 2018 Jun 26.
3. Sani G, Napoletano F, Vöhringer PA, Sullivan M, Simonetti A, Koukopoulos A, Danese E, Girardi P, Ghaemi N. Mixed depression: clinical features and predictors of its onset associated with antidepressant use. Psychother Psychosom. 2014;83:213–21.
4. Sani G, Vöhringer PA, Barroilhet SA, Koukopoulos AE, Ghaemi SN. The Koukopoulos Mixed Depression Rating Scale (KMDRS): An International Mood Network (IMN) validation study of a new mixed mood rating scale. J Affect Disord. 2018;232:9–16.
5. Sani G, Vöhringer PA, Napoletano F, Holtzman NS, Dalley S, Girardi P, Ghaemi SN, Koukopoulos A. Koukopoulos׳ diagnostic criteria for mixed depression: a validation study. J Affect Disord. 2014;164:14–8.

## A22 Fostering adaptive stress management via technology-mediated mindfulness practice: self-report and psychophysiological evidence

### Michela Balconi^1,2^, Giulia Fronda^1,2^, Irene Venturella^1,2^, Davide Crivelli^1,2^

#### ^1^Research Unit in Affective and Social Neuroscience, Catholic University of the Sacred Heart, Milan, Italy; ^2^Department of Psychology, Catholic University of the Sacred Heart, Milan, Italy

##### **Correspondence:** Giulia Fronda - giulia.fronda@unicatt.it

*Annals of General Psychiatry* 2018, **17(Suppl 1):**A22

**Background:** Several studies have recently observed the efficacy of mindfulness practices in individuals’ psychophysical well-being and stress management [1]. Mindfulness suggested by traditional approaches are based on the acceptance of one’s own mental states and require individual constant commitment and an intense exercise, which usually inhibit beginners. Whereas research have shown the usefulness of body-sensing devices, that are able to control and provide a feedback on meditation progress, in reducing the negative impact of traditional approaches demand [2,3]. So, the potential effects of a mindfulness intervention, supported by using a wearable device, were investigated considering results on stress management and subjective well-being evaluation.

**Materials and Method:** A 4-week mindfulness intervention consisting of daily sessions was tested on forty participants, divided randomly into an active control and experimental group. Experimental group participants were asked to perform daily mindfulness practices supported by the use of a wearable device with an embedded EEG recording system. To evaluate the mindfulness practice effects, physiological and subjective stress-related markers were collected before and after intervention.

**Results:** Results highlighted the efficacy of mindfulness intervention on stress levels decrease for the experimental group participants compared to controls. Specifically, an improvement in physiological markers and parasympathetic activity (standard deviation of inter-beat intervals) was observed during resting-state condition and cognitive stressful performance.

**Conclusions:** These findings suggested the efficacy of an intensive technology-mediated mindfulness intervention in the enhancement of subjective well-being and in stress management, demonstrated by a decrease of anxiety and an increased control on stressful events.

**Ethics Approval:** This study was approved by the Ethics Committee of the Department of Psychology of the Catholic University of the Sacred Heart of Milan, Italy.

**Consent to publish:** Written informed consent for publication was obtained from all participants.

**References**

1. Siegel DJ. Mindfulness training and neural integration: Differentiation of distinct streams of awareness and the cultivation of well-being. Soc Cogn Affect Neurosci. 2007; 2:259–63.
2. Balconi M, Fronda G, Venturella I, Crivelli D. Conscious, pre-conscious and unconscious mechanisms in emotional behaviour. Some applications to the mindfulness approach with wearable devices. Appl Sci. 2017; 7:1280.
3. Balconi M, Crivelli D. Wearable devices for self-enhancement and improvement of plasticity: effects on neurocognitive efficiency. In: Esposito A, Cordasco G, editors. Smart Innovation, Systems and Technologies: Dynamics of Signal Exchanges. Heidelberg: Springer. *In Press*.

## A23 Which are the most frequent comorbidities of alcohol dependence in women in Southern Bulgaria?

### Desislava Ivanova^1^, Vaitsa Giannouli^2^

#### ^1^Department of Psychology, South-West University “Neofit Rilski”, Blagoevgrad, Bulgaria; ^2^Organisation Against Drugs, OKANA, Greece

##### **Correspondence:** Vaitsa Giannouli

*Annals of General Psychiatry* 2018, **17(Suppl 1):**A23

**Background:** Although, the presence of one or more additional diseases or disorders are widely known for alcohol dependence, the comorbidity profile of alcohol dependent women is not examined so far in detail. The aim of this study is to examine which are the most prevalent comorbidities in a sample of alcohol dependent women in Southern Bulgaria.

**Method:** Fifty-three women with a diagnosis of alcohol dependence (M_age_ = 43.84, SD_age_ = 9.48; M_years ofeducation_ = 15.11, SD _years of education_ = 3.21; M_years of addiction_12, SD_years of addiction_ = 3.56) were examined with the Bulgarian version of the Lesch Alcoholism Typology − Questionnaire (LAT), while data were collected for their physical and mental comorbidities during their examination and therapy sessions at the Municipal Council on Drug Addiction in Blagoevgrad.

**Results:** Descriptive statistics showed physical comorbidity of alcohol dependence with gastrointestinal diseases 43.39% (N = 23), and comorbidity of alcoholism and psychiatric disorders such as mood disorders 15.09% (Ν = 8) and parallel other than alcohol substance abuse 13.20% (N = 7). The rates of comorbidity (or dual diagnosis) with affective disorders is much more common compared to the results in foreign studies or the comorbidity data regarding other diseases in women.

**Conclusions:** According to the above results, women with alcohol dependence have high rates of comorbidities and do experience a wide range of comorbid diseases, thus making the therapy process a complicated and multi-faceted procedure. Interestingly, alcohol dependence is more prevalent in anxious and/or depressed persons.

**Ethics approval:** The study was approved by Municipal Council on Drug Addiction in Blagoevgrad Ethics Board.

## A24 Fake news: is it a social phenomenon based on neuropsychologically determined self- or other-deceptive mechanisms? Some thoughts based on insight and self-awareness’ areas

### Orestis Giotakos

#### Obrela, 2 Erifilis, 11634 Athens, Greece

##### **Correspondence:** Orestis Giotakos - info@obrela.gr

*Annals of General Psychiatry* 2018, **17(Suppl 1):**A24

*Fake news* is intentionally misleading and deceptive news that is written and published with the intent to damage an entity or a person. Fake news is a type of yellow journalism that consists of deliberate misinformation or hoaxes spread via traditional print or online social media. They may contain false, misleading, imposter, manipulated or fabricated content.

In neuropsychiatry, the concept of *self*- *or other*-*deception* is defined as the eclectic and self-motivated absence of understanding of material that is psychologically comprehensible. It has been theorized as a *deviant expansion of insight or self*-*awareness*. The following process describe the phenomenon of self-deception: (a) the person has two mental contents, that are conflicting when expressed as propositions, (b) these two mental contents occur simultaneously, (c) the person is not aware of one of the two mental contents, (d) the process that defines which mental content is subject to awareness depends on the individual’s motivation [1]. Multiple neuropsychological theories of awareness emphasize the role of an *error*-*monitoring system,* which is primarily linked with the *hippocampal system*, while other brain areas involved are the *dorsolateral prefrontal cortex*, the *supplementary motor area* and the *anterior cingulate gyrus* [2].

People with high levels of self-deception simply deny or minimize their perception of symptoms, negating the presence of symptoms primarily to themselves. This absence of denial could be conceptualized as a form of *depressive realism*. Conversely, *good insight* can be viewed as an example of “depressive realism”. Higher levels of self-deception are associated with less or less severe symptoms of depression. The phenomenon of self-awareness in affective disorders can be viewed on a continuum, with severe depression occupying one end, followed by mildly depressed patients with low levels of self-deception (the so-called *depressive realism*), and in the other end of the spectrum a group of healthy individuals with high levels of self-deception. Interestingly, this continuum seems to be independent of the psychotic process [1, 3].

In conclusion, *self*-*deception process* may have a protective role against depression, since patients with poor insight manifest less depressive symptoms, while depression on its own may reduce mechanisms of self-deception. We may suggest that underlined neuropsychological processes, probably based on biologically determined self- or other-deceptive mechanisms, could serve in the development, and even the conservation, of at least some of the social behaviors related to the fake news phenomenon.

1. Sackeim, H. A. Self-deception: A synthesis. In J. S. Lockard, & D. L. Paulhus (Eds.), *Self*-*deception: An adaptive mechanism*, New Jersey: Prentice Hall, 1988, 146–165.

2. Petrides, M. The mid-dorsolateral prefronto-parietal network and the epoptic process. In D.T. Stuss & R.T. Knight (eds), Principles of Frontal Lobe Function, Chapter 7. New York: Oxford University Press, 2013, 79–89.

3. David A, Bedford N, Wiffen B, Gilleen J. Failures of metacognition and lack of insight in neuropsychiatric disorder. Phil. Trans. R. Soc. B., 2012, 367: 1379–1390.

## A25 ICD symptomatology in Parkinson’s Disease: first data from a cohort of patients

### Alexandros Kapsomenakis, Dimitrios S. Kasselimis, Marianthi Breza, Georgios Konstantakopoulos, Constantin Potagas

#### National and Kapodistrian University of Athens, Athens, Greece

##### **Correspondence:** Alexandros Kapsomenakis - Kapsomenakis.a@gmail.com

*Annals of General Psychiatry* 2018, **17(Suppl 1):**A25

**Background:** Several studies have described -among other non-motor symptoms in Parkinson’s Disease (PD)- Impulse Control Disorder symptomatology. The aim of the present study is to explore the prevalence of these symptoms in a large cohort of patients.

**Materials and Methods:** This is a retrospective study. The original patient pool consists of 861 PD patients examined at the Specialized Movement Disorders outpatient clinic of the First Neurology Department of the National and Kapodistrian University at Eginition Hospital. A case-by-case investigation was performed to identify patients with non-motor symptoms, which were then classified in several categories. Comorbidity with other neurological and previous psychiatric disorders, or limited number of visits were considered as exclusion criteria. This study has been approved by the Eginition Hospital Ethics Committee.

**Results:** One hundred and four were identified with at least one ICD, out of which 31 manifested more than one ICDs. The most frequent ICD (64 patients) was food-related (binge eating). Thirty patients demonstrated compulsive buying, 23 patients exhibited gambling behavior, and 14 patients manifested hypersexuality.

**Conclusions:** ICDs are shown to be common among a subgroup of PD patients demonstrating non-motor, psychiatric symptomatology. Such symptoms may affect the patients’ course (for example, patients’ compliance to treatment and overall co-operation). Relationships between ICD symptomatology, clinical and demographic variables, as well as type and duration of pharmacological treatment, are also discussed.

## A26 Esketamine in rapid response and long term treatment of treatment-resistant depression?

### Kasper Siegfried

#### Department of Psychiatry and Psychotherapy, Medical University of Vienna, Vienna, Austria

##### **Correspondence:** Kasper Siegfried - sci-genpsy@meduniwien.ac.at

*Annals of General Psychiatry* 2018, **17(Suppl 1):**A26

Clinical trials demonstrated that ketamine exhibits rapid antidepressant efficacy when administered in subanesthetic dosages. We reviewed currently available literature investigating efficacy, response rates and safety profile.

Twelve clinical trials investigating unipolar depressed patients were included after systematic literature search. Additionally literature in bipolar patients was reviewed.

Antidepressant response rates on primary outcome measures after 24 h were 61% (average) The average reduction of Hamilton Depression Rating Scale (HDRS) was 10.9 points, Beck Depression Inventory (BDI) 15.7 points and Montgomery-Asberg Depression Rating Scale (MADRS) 20.8 points. Ketamine was always superior to placebo. Most common side effects were dizziness, blurred vision, restlessness, nausea/vomiting and headache, which were all reversible. Relapse rates ranged between 60% and 92%. Based on these findings, a consent-form for clinical application and modification in local language is included as supplementary material.

Ketamine constitutes a novel, rapid and efficacious treatment option for patients suffering from treatment-resistant depression and exhibits a rapid and significant anti-suicidal effect. New administration routes might serve as alternative to intravenous regimes for potential usage in outpatient settings. However, limited duration of treatment response with high relapse rates within the first month after treatment demand for developments to prolong ketamine’s efficacy.

## A27 Repetitive transcranial magnetic stimulation (rTMS) in the treatment of bipolar depression: preliminary data

### Monika Klírová^1,2^, Tomáš Novák^1,2^, Veronika Voráčková^1^, Lenka Kostýlková^1,2^, Martin Hejzlar^1,2^, Jiří Renka^1,2^, Silvie Čerešňáková^1^

#### ^1^National Institute of Mental Health, Klecany, 250 67, Czech Republic; ^2^3rd Faculty of Medicine, Charles University, Prague, 100 00, Czech Republic

##### **Correspondence:** Monika Klírová - monika.klirova@nudz.cz

*Annals of General Psychiatry* 2018, **17(Suppl 1):**A27

**Background:** There is a significant lack of effective treatment strategies for bipolar depression (BDE) [1]. While the efficacy of repetitive transcranial magnetic stimulation (rTMS) in major depression disorder (MDD) is well established [2], there is a substantially less evidence supporting its efficacy in BDE [3]. Based on imaging studies [4], stimulation sites routinely used in MDD, specifically left and right dorsolateral prefrontal cortex (DLPFC) might not be the best choice for BDE. Instead, the ventrolateral PFC (VLPFC) abnormalities have been found frequently in patients with BDE.

**Materials and Methods:** A 4-week, randomized, sham-controlled, double-blind, three-group parallel study with T1 weighted MRI-guided rTMS was used. Patients were randomly allocated to the 3 intervention groups: active rTMS applied to the a) left DLPFC, b) right VLPFC, and c) sham rTMS. Stimulation parameters were: 10 Hz, 100% of motor threshold, 2 s on, 8 s off, 10 min duration; 1200 pulses/session, 20 sessions. Patients were clinically assessed by Montgomery and Åsberg Rating Scale (MADRS) and Young Mania Rating Scale (YMRS) at baseline and weekly during the 4 weeks of treatment.

**Results:** 23 out of 25 enrolled patients were included into Intention-to-treat (ITT) analysis. Two patients drop out within first week (one on DLPFC and one on VLPFC). The final sample was 23 (8 on VLPFC, 7 on DLPFC, 8 on sham). The treatment groups did not differe in age, gender, duration of episodes, as well as in baseline MADRS and YMRS. Both active rTMS shown better improvement (as assessed by MADRS) compared to sham rTMS after 4 weeks. The standardized difference was moderate in VLPFC (Cohen´s d = 0.75) and only small in DLPFC (d = 0.30). The response rate across the sample was 35%, and there was no difference in response rates across the groups. YMRS remained unchanged within the study time (2.2–3.4) and no switch to hypomania were observed.

**Conclusions:** Our preliminary data confirmed the efficacy of active rTMS protocols in the treatment of BDE. rTMS of right VLPFC has shown better improvement then rTMS of the left DLPFC. Our results are in agreement with actual findings from imaging studies in patients with BDE [4] and support the idea, that right VLPFC plays an important role in the neurobiology of BDE.

**Acknowledgements:** Study was funded by the Ministry of Health of the Czech Republic grant nr. 16-31380A.

**References**

1. Sienaert P, Lambrichts L, Dols A, De Fruyt J. Evidence-based treatment strategies for treatment resistant bipolar depression: a systematic review. Bipolar Disord. 2013; 15(1):61–9.
2. Lefaucheur JP, Andre-Obadia N, Antal A, et al. Evidence-based guidelines on the therapeutic use of repetitive transcranial magnetic stimulation (rTMS). Clin Neurophysiol. 2014; 125(11):2150–206.
3. Nahas Z, Kozel FA, Li X, Anderson B, George MS. Left prefrontal transcranial magnetic stimulation (TMS) treatment of depression in bipolar affective disorder: a pilot study of acute safety and efficacy. Bipolar Disord. 2003; 5(1):40–7.
4. Chen CH, Suckling J, Lennox BR, Ooi C, Bullmore ET. A quantitative meta-analysis of fMRI studies in bipolar disorder. Bipolar Disord. 2011; 13(1):1–15.

## A28 The Koukopoulos Mixed Depression Rating Scale (KMDRS): a new tool for an old syndrome

### Alexia Koukopoulos^1,2^, Lavinia De Chiara^3^, Giovanni Manfredi^2,3^, Gloria Angeletti^2,3^, Gabriele Sani^2,3^

#### ^1^Umberto I Hospital, Psychiatry Department, Rome, Italy; ^2^Centro Lucio Bini, Rome, Italy; ^3^NeSMOS Department, Sapienza University of Rome, Italy

##### **Correspondence:** Alexia Koukopoulos - alexiakoukopoulos@gmail.com

*Annals of General Psychiatry* 2018, **17(Suppl 1):**A28

**Background:** From classical antiquity, melancholia was described in various forms, many of which would today be considered mixed affective states (as Hippocrates (460–377 A.C), Aretaeus of Cappadocia in the 1st century AD, Ibn Imran in the 10th century AD) [1]. Also the classical authors in modern western phychiatry, until the end of the nineteenth century continued to replicate these ancient truths (as JCA. Heinroth, B. Rush, W. Griesinger, JP. Falret, J. Baillarger, F. Richarz, E. Kraepelin, W. Weygandt) [2]. On the contrary the DSM system, since the first edition in 1952, has placed mixed features in a peripheral position making it difficult for clinicians to diagnose mixed depressive states and having an enormous impact on therapeutic approaches. In a series of papers by our group we have presented the hypothesis that the DSM-5 criteria for mixed depression have poor clinical utility and suggest that an alternative set of symptoms could be adopted to make the diagnosis of mixed depression possible [3]. Athanasios Koukopoulos published criteria for Mixed Depression (MxD) which encompass clinical pictures characterized by marked psychomotor or inner excitation and rage/anger, along with severe depression [4]. The KMDRS is the first rating scale specifically designed to assess MxD. It was developed in order to enable clinicians and research investigators to collect data assessing the presence and severity of symptoms of excitatory or mixed nature in individuals diagnosed as suffering from a Major Depressive Episode (MDE) according to the DSM-IV criteria. It is comprised of 14 items, designed to be administered by a trained clinician, and was recently validated [5].

**Methods**: 350 patients from the international mood network (IMN) completed three rating scales: the KMDRS, Montgomery-Asberg Depression Rating Scale (MADRS) and Young Mania Rating Scale (YMRS). KMDRS’ psychometric properties assessed included Cronbach’s alpha, inter-rater reliability, factor analysis, predictive validity, and Receiver Operator Curve analysis.

**Results**: Internal consistency (Cronbach’s alpha = 0.76; 95% CI 0.57, 0.94) and interrater reliability (kappa = 0.73) were adequate. Confirmatory factor analysis identified 2 components: anger and psychomotor excitation (80% of total variance). Good predictive validity was seen (C-statistic = 0.82). Severity cut-off scores identified were as follows: no (0–4), possible (5–9), mild (10–15), moderate (16–20) and severe (> 21) MxD.

**Limitations:** Non DSM-based diagnosis of MxD may pose some difficulties in the initial use and interpretation of the scoring of the scale. Moreover, the cross-sectional nature of the evaluation does not verify the long-term stability of the scale.

**Conclusions**: KMDRS was a reliable and valid instrument to assess MxD symptoms.

**References**

1. Koukopoulos A, Sani G, Koukopoulos AE, Manfredi G, Pacchiarotti I, Girardi P. Melancholia agitata and mixed depression. Acta Psychiatr Scand suppl. 2007;(433):50–7.
2. Koukopoulos A, Albert MJ, Sani G, Koukopoulos AE, Girardi P. Mixed depressive states: nosologic and therapeutic issues. Int Rev Psychiatry. 2005;17:21–37.
3. Koukopoulos A, Sani G. DSM-5 criteria for depression with mixed features: a 4farewell to mixed depression. Acta Psychiatr Scand. 2014;129:4–16.
4. Sani G, Vöhringer PA, Napoletano F, Holtzman NS, Dalley S, Girardi P, Ghaemi SN, Koukopoulos A. Koukopoulos׳ diagnostic criteria for mixed depression: a validation study. J Affect Disord. 2014;164:14–8.
5. Sani G, Vöhringer PA, Barroilhet SA, Koukopoulos AE, Ghaemi SN. The Koukopoulos Mixed Depression Rating Scale (KMDRS): An International Mood Network (IMN) validation study of a new mixed mood rating scale. J Affect Disord. 2018;232:9–16.

## A29 Expression of COMT ALDH1A1, MAO-A, MAO-B in PC 12 molecular series under various vitamin D concentrations, in vitro experimental findings

### Alexandros Mitrusias

#### Department of Molecular Biology and Genetics, School of Health Sciences, Democritus University of Thrace, Alexandroupolis, Greece

##### **Correspondence:** Alexandros Mitrusias

*Annals of General Psychiatry* 2018, **17(Suppl 1):**A29

The experimental process (materials, steps and reason for conducting each sub-process) that was carried out to conduct the research is mentioned below.

In particular, the cell-splitting process is carried out to stabilize and normalize the growth rate of cells when their density in the flask reaches approximately 70%–100%. Moreover, the process of clotting the cells into 100 Petri dishes, which targets both the counting of cells and the procedure of the effect of Vitamin D on PC-12 cells, is reported.

Then, the procedure of “pickling” the cells that have undergone the above effect is performed so that they are placed in small falcons to effect the extraction of RNA from the cells. This process aims to extract the mRNA from the genes expressed in our cells in order to synthesize the cDNA and perform quantitative analysis of gene expression via real-time PCR. However, in between the procedures of RNA extractions and cDNA synthesis, NanoDrop quality control has been mediated to help us understand whether RNA extraction has been substantiated as it gives us information about the concentration of mRNA in each of the falcons used (information very important to determine, as well, the amounts of water we need in cDNA synthesis). We also mention the reason why we chose real-time PCR procedure, the characteristics of this reaction, and the steps taken to complete the process.

Finally, the expressions of the four genes is presented quantitatively, (at all the concentrations of vitamin D used in the effects) as well as the photographic material from the electronic microscopes of the affected cells (at the various concentrations and in different zooming enchantments) so that we have both quantitative results as well as morphological, developmental and popliteal information for these cells.

## A30 Neuropsychological functioning in child and adolescent offspring of parents with bipolar disorder

### Tomas Novak^1^, Antonin Sebela^1^, Marketa Mohaplova^2^, Michaela Viktorinova^1^, Michal Goetz^2^

#### ^1^National Institute of Mental Health, Klecany, Czech Republic; ^2^Department of Paediatric Psychiatry, Motol University Hospital, Prague, Czech Republic

##### **Correspondence:** Tomas Novak

*Annals of General Psychiatry* 2018, **17(Suppl 1):**A30

**Background**: Although positive family history is the strongest predictor for bipolar disorder (BD), most offspring of parent with BD (OBP) will not develop the illness. Therefore, an identifying markers of vulnerability is essential for the individual risk estimation. Besides a number of candidates, worse cognitive performance is considered to be a plausible marker of risk for BD development.

**Materials and methods:** Sixty OBP (47% girls; 11.7 ± 3.2 years) and 42 control offspring (offspring of healthy parents; OHP) with comparable gender proportion (45% girls, p = .89) and age (12.4 ± 3.0, p = .27), were assessed using the Kiddie Schedule for Affective Disorders and Schizophrenia (K-SADS) for presence of DSM-5 current and life-time diagnoses. The General Behavior Inventory -Parent version (GBI-P) was applied to detect subthreshold mood symptoms. Subsequently, they were tested for intelligence, verbal fluency and memory, psychomotor speed, attention, executive functions and social cognition by Raven´s Progressive Matrices, the Developmental Neuropsychological Assessment battery (NEPSYII), the D2 test of Attention, and the Amsterdam Neuropsychological Tasks (ANT).

**Results:** Thirty-eight of OBP (63%) and 8 controls (19%) met the criteria for at least one current DSM-5 diagnosis with anxiety disorders as the most frequent diagnosis and 17 (28%) OBP and 1 OHP (2%) exceeded GBI cut-off for subthreshold mood symptoms. Despite extensive morbidity and current mood symptoms in OBP, groups did not differ in intelligence (115.2 ± 14.4 vs 116.5 ± 14.2; p = .65), neither in performance in any test of whole neuropsychological battery (Hedge´s g ranged -0.21 to 0.39). Even a subsequent comparison of OBP with mood symptoms and gender-and age-matched OHP failed to find significant differences.

**Conclusions:** We failed to find difference in performance across broad cognitive battery between high-risk and control offspring. Contrary to some of the previous findings, we cannot confirm that cognitive performance might be a promising marker or endophenotype for bipolar disorder.

**Acknowledgements:** Study was funded by the Ministry of Health of the Czech Republic grant nr. 17-32478A.

## A31 Interpretation of the experimental findings of COMT ALDH1A1, MAO-A, MAO-B expression in PC 12 molecular series under various vitamin D concentrations

### Aglaia Pappa

#### Department of Molecular Biology and Genetics, School of Health Sciences, Democritus University of Thrace, Alexandroupolis, Greece

##### **Correspondence:** Aglaia Pappa

*Annals of General Psychiatry* 2018, **17(Suppl 1):**A31

Vitamin D is the key ingredient of the effects on the experimental procedures we described above. For that reason we first mention its peculiarities, with particular emphasis on the crucial role it plays in brain development as well as the onset of disorders, possibly due to its lack. In addition, the properties and characteristics that have been a key determinant of PC-12 cell line are highlighted. Plus, the genes studied in this procedure (COMT, MAO-a, MAO-b, ALDH1A1) are described.

The results are in accordance with the current literature. Genes have their optimum expression in the normal concentrations of Vitamin D, which also seems to promote PC-12 differentiation.

However modest the results of this experiment might be there are interesting especially for clinicians who might acquire deeper knowledge for the role of Vitamin D in dopamine metabolism.

It is also to our view quite satisfactory the fact that there was fruitful collaboration between the Department of Medicine and the Department of Molecular Biology and Genetics within the context of School of Medical Sciences of Democritus University of Thrace. This collaboration can work as pilot collaboration that can lead to further joint research work we beneficiary effects for all the involved parties.

## A32 Agitated/mixed depression: the clinical controversy

### Gabriele Sani^1,2^, Lavinia De Chiara^1^, Giovanni Manfredi^1,2^, Gloria Angeletti^1,2^, Alexia Koukopoulos^2,3^

#### ^1^NeSMOS Department, Sapienza University of Rome, Rome, Italy; ^2^Centro Lucio Bini, Rome, Italy; ^3^Umberto I Hospital, Psychiatry Department, Rome, Italy

##### **Correspondence:** Gabriele Sani - gabriele.sani@uniroma1.it

*Annals of General Psychiatry* 2018, **17(Suppl 1):**A32

**Background**: Many authors clearly described mixed states well before Kraepelin. Kraepelin conceptualized and described mixed states in a systematic way. In conceiving the manic-depressive mixed states, Kraepelin started from the excitement or depression of the three domains of psychic life: the intellect (train of thought rather than its contents), mood, and volition, expressed in psychomotor activity. He made them the cornerstone of the manic-depressive entity [1]

**Methods**: An extensive historical search for mixed depression was performed. Furthermore, a systematic review of the papers used by DSM-5 as reference papers, and a PubMed search were performed.

**Results:** Interest in mixed states was waning by the 1920s. The American Psychiatry Association, through the DSM system, placed the mixed states in a peripheral position. Now the DSM-5 proposed the “mixed features” which could be applied to either major depressive disorder or bipolar disorder. This is certainly a good position. The problem is that the so called overlapping symptoms, i.e. those symptoms that can be present both in depressive and (hypo)manic episodes, are, by definition, excluded from the mixed features [2]. In recent years, a growing number of psychiatrists have expressed disenchantment with the official view. In fact, symptoms like psychic and motor agitation, racing or crowded thoughts, irritability or unprovoked feelings of rage, talkativeness, mood lability and insomnia are clearly symptoms of nervous excitability and they constitute the essence of the mixed affective episode. Alternative diagnostic criteria have been proposed [3].Their exclusion, based on a theoretical choice and not on clear clinical evidence, makes the diagnosis of these patients difficult leading the clinicians to inadequate treatments. A specific scale to help clinicians and researchers to better diagnose mixed depression have been created [4].

The adverse response of these states to antidepressant drugs, above all the increase of agitation and of suicidality, makes a clear distinction between simple and mixed depression necessary and urgent. The suicidality induced by antidepressants is related to manifest or latent agitation. Latent agitated depression will be introduced. In mixed depression, treatment should initiate with anti-psychotics, anti-epileptics, usually at low doses preferably according to dose serum [5,6] lithium and benzodiazepines and when agitation has subsided, and if simple depression persists, antidepressants may be used cautiously. Electroconvulsive therapy is very effective throughout the course of agitated depression [1].

**Limitation:** This presentation carries the limitation of a conceptual review.

**Conclusions**: In our clinical practice, we have seen the 3rd and 4th criteria (namely, more talkative than usual or pressure to keep talking, and flight of ideas or subjective experience that thoughts are racing) frequently in mixed depression, but the other five criteria are extremely rare, if ever present.

On this topic of mixed features, DSM-5 is taking the psychiatric world down a path of less and less scientific evidence, and in this paper, we plan to show how this is the case, and how wrong the above definition is, and how harmful it will be to those unfortunate people who suffer from the mixed depression that DSMs III through 5 have refused to see.

**References**

1. Koukopoulos A, Sani G, Koukopoulos AE, Manfredi G, Pacchiarotti I, Girardi P. Melancholia agitata and mixed depression. Acta Psychiatr Scand suppl. 2007;(433):50–7.
2. Koukopoulos A, Sani G. DSM-5 criteria for depression with mixed features: a farewell to mixed depression. Acta Psychiatr Scand. 2014;129:4–16.
3. Sani G, Vöhringer PA, Napoletano F, Holtzman NS, Dalley S, Girardi P, Ghaemi SN, Koukopoulos A. Koukopoulos׳ diagnostic criteria for mixed depression: a validation study. J Affect Disord. 2014;164:14–8.
4. Sani G, Vöhringer PA, Barroilhet SA, Koukopoulos AE, Ghaemi SN. The Koukopoulos Mixed Depression Rating Scale (KMDRS): An International Mood Network (IMN) validation study of a new mixed mood rating scale. J Affect Disord. 2018;232:9–16.
5. Musenga A, Saracino MA, Sani G, Raggi MA. Antipsychotic and antiepileptic drugs in bipolar disorder: the importance of therapeutic drug monitoring. Curr Med Chem. 2009;16:1463–81.
6. Lostia AM, Mazzarini L, Pacchiarotti I, Lionetto L, De Rossi P, Sanna L, Sani G, Kotzalidis GD, Girardi P, Simmaco M, Tatarelli R. Serum levels of risperidone and its metabolite, 9-hydroxyrisperidone: correlation between drug concentration and clinical response. Ther Drug Monit. 2009;31:475–81.

## A33 Instrumental registration of specific oculomotor biomarkers in patients with schizophrenia

### Kalin Stoynov, Toni Donchev

#### ^1^Medical Center Intermedica, Sofia, Sofia, Bulgaria

##### **Correspondence:** Kalin Stoynov - kalin_stoynov_@abv.bg

*Annals of General Psychiatry* 2018, **17(Suppl 1):**A33

**Background:** There are uncertainties in etiological and pathophysiological aspects of schizophrenia leading to limitations in the early diagnosis and treatment. A study approach towards the endophenotypes provides limited biological markers as a gene expression of certain disorder rather than the clinical phenotype [1]. Endophenotypes are measurable components, invisible to the bare eye, located between the visible manifestations and the genotype and appear to be an important concept in the study of complex neuropsychiatric disorders [1]. Dysfunction of the smooth pursuit eye movements of objects has long been connected with schizophrenia [2].

**Materials and methods:** In this study the investigated subjects are divided into two groups. The first consists of 107 patients with schizophrenia and the second one is a control group of 110 healthy subjects. An instrumental method for registration of smooth pursuit eye movements was used. One of the device modules includes detection of horizontal eye movements characteristics—frequencies measured in Hz.

**Results:** In this prospective study the eye movements of investigated groups are compared by the average frequencies, detected by device during the first 30 s of the smooth pursuit. It was found out the average frequency in the group of schizophrenic patients was 2.013 Hz. In comparison 0.771 Hz was the average frequency measured in the control group subjects.

**Conclusions:** There is a significant difference between the two studied groups. The relationship between the registered changes in smooth pursuit eye movements and schizophrenia is evident—2.6 times higher average frequency which could be considered as a reliable biomarker in schizophrenic patients.

**References**

1. Gottesman I, Gould T. The Endophenotype Concept in Psychiatry: Etymology and Strategic Intention. Am J Psychiatry 2003; 160:636–645.
2. Thaker G. Neurophysiological Endophenotypes Across Bipolar and Schizophrenia Psychosis. Schizophr Bull 2008; 34(4):760–773.

## A34 Data processing validation of predictive cluttering inventory through a pilot study in typical Greek population

### Maria Cherkeletzi^1^, Eleni Kapeta^1^, Soultana Soglemezi^1^, Evangelia I. Kosma^2,3^, Spyridon K. Chronopoulos^4,5^, Dionysios Tafiadis^1^

#### ^1^Department of Speech & Language Therapy, Technological Educational Institute of Epirus, Ioannina, Greece; ^2^Faculty of Medicine, School of Health Sciences, University of Thessaly, Biopolis, Larissa, Greece; ^3^Psychologist, Private Practice, Mihail Aggelou 18, Ioannina, Greece; ^4^Department of Informatics and Telecommunications Engineering, University of Western Macedonia, Kozani, Greece; ^5^Department of Computer Engineering, Technological Educational Institute of Epirus, Arta, Greece

##### **Correspondence:** Dionysios Tafiadis - d.tafiadis@ioa.teiep.gr; tafiadis@gmail.com

*Annals of General Psychiatry* 2018, **17(Suppl 1):**A34

**Background:** Cluttering is a fluency disorder characterized by an abnormally rapid rate. Several studies about its diagnosis were conducted based on different processes, such as articulation’s and phonological assessments and/or collection of spontaneous speech samples. Standardized diagnostic tools had not been proposed till Davis et al. suggested the “Predictive Cluttering Inventory (PCI)” as a self-perceived questionnaire for adults. The purpose of this study is to validate PCI in typical Greek adult population.

**Materials and methods:** The translated version of PCI was administrated in 361 typical adults (180 male and 181 female) aged from 18 το 87 years of age (M = 49.10, SD = 18.60). The questionnaire was adjusted to Greek language according to the minimum translation criteria.

**Results:** No statistical significant differences were observed between male and female on PCI’s total score (U = 16264.500, NS) and its four domains. On the contrary, statistical significant differences was calculated for PCI total score for all age subgroups (Η = 58.692, p = .000). Furthermore, the averages scores of the younger aged subgroups had lower scores in comparison to the averages scores of older aged ones. PCI questionnaire had high internal consistency (Reliability Coefficients 33 items Alpha = .972).

**Conclusions:** PCI questionnaire appears to be proper for standardization to adults with cluttering in its current form. It also discriminated well how adults of all ages understood the characteristics of this fluency disorder. Further research must be conducted for processing more evidenced based data and clinical use as in other disorders [1–9] for mental health professionals.

**References**

1. Fountoulakis K N, Iacovides K, Kleanthous S, Samoilis S, S Kaprinis G, Sitzoglou K, Bech P: Reliability, validity and psychometric properties of the Greek translation of the Center for Epidemiological Studies-Depression (CES-D) Scale. BMC Psych 2001, 1(2): 3
2. Tafiadis D, Chronopoulos S K, Kosma E I et al. Using Receiver Operating Characteristic Curve to Define the Cutoff Points of Voice Handicap Index Applied to Young Adult Male Smokers. Journal of Voice. 2018;32(4):443–448. 10.1016/j.jvoice.2017.06.007
3. Tafiadis D, Kosma E I, Chronopoulos S K et al. Voice Handicap Index and Interpretation of the Cutoff Points Using Receiver Operating Characteristic Curve as Screening for Young Adult Female Smokers. Journal of Voice. 2018;32(1):64–69. 10.1016/j.jvoice.2017.03.009
4. Tafiadis D, Chronopoulos S, Siafaka V et al. Comparison of Voice Handicap Index Scores Between Female Students of Speech Therapy and Other Health Professions. Journal of Voice. 2017;31(5):583–588. 10.1016/j.jvoice.2017.01.013
5. Tafiadis D, Kosma E, Chronopoulos S et al. Acoustic and Perceived Measurements Certifying Tango as Voice Treatment Method. Journal of Voice. 2018;32(2): 256.e13–256.e24. 10.1016/j.jvoice.2017.05.016
6. Voniati L. School Based Speech and Language Therapists’ Perceptions of MLU-w and Its Use. Science Journal of Education. 2015; 3(6):119–125. 10.11648/j.sjedu.20150306.11
7. Voniati L. Mean Length of Utterance in CYG-speaking children. Journal of Greek Linguistics. 2016; 16(1): 117–140.
8. Voniati L. Christopoulou M. Developing an Augmentative and Alternative Communication System for a Child with Autism Spectrum Disorder: A Case Study, American Journal of Health Research. 2017; 1(2): 58–62. 10.11648/j.ijcsd.20170102.15
9. Voniati L. Charalambous, I. Rett syndrome: Clinical recognition, communication skills and therapeutic intervention, Archives of Hellenic Medicine. 2018; 35(2): 188–197.

**Ethics Approval:** The study was approved by the Ethical Committee of Department of Speech Language Therapy (School of Health and Welfare Professions) TEI of Epirus

**Consent to publish:** Informed consent to publish has been obtained from each participant.

## A35 Perceived levels of fluency, statistically analyzed for typical male adults using predictive cluttering inventory: a pilot study in Greek population

### Maria Cherkeletzi^1^, Eleni Kapeta^1^, Soultana Soglemezi^1^, Evangelia I. Kosma^2,3^, Spyridon K. Chronopoulos^4,5^, Dionysios Tafiadis^1^

#### ^1^Department of Speech & Language Therapy, Technological Educational Institute of Epirus, Ioannina, Greece; ^2^Faculty of Medicine, School of Health Sciences, University of Thessaly, Biopolis, Larissa, Greece; ^3^Psychologist, Private Practice, Mihail Aggelou 18, Ioannina, Greece; ^4^Department of Informatics and Telecommunications Engineering, University of Western Macedonia, Kozani, Greece; ^5^Department of Computer Engineering, Technological Educational Institute of Epirus, Arta, Greece

##### **Correspondence:** Dionysios Tafiadis - d.tafiadis@ioa.teiep.gr; tafiadis@gmail.com

*Annals of General Psychiatry* 2018, **17(Suppl 1):**A35

**Background:** Fluency disorders and its etiologies was always of interest by mental health professionals. Cluttering -as a fluency disorder- is a clinical condition that needs to be diagnosed accurately and extensively. Self-perceived questionnaires are a new trend in evidence-based assessment, and gender is a factor that influences their results. Particularly, “Predictive Cluttering Inventory (PCI)” is a questionnaire that elicits information from adults with cluttering disorders. Purpose of the current study was to extract evidence-based data from typical male population using PCI.

**Materials and methods:** The validated PCI (in Greek language) was administered to 180 typical Greek adult males aged from 18 to 86 (M = 49.12, SD = 18.514). Participants that met criterions such as psychiatric/neurogenic disorders or having been illiterate were excluded from this study.

**Results:** Statistical significant differences were observed between all age groups for PCI’s total score [Η(5) = 21.299, p = .001] for male participants. Similarly, statistical significant differences were calculated between 70 + age subgroup and the rest age subgroups 18–30 (U = 193.000, p = .000), 30–40 (U = 230, p = .000), 40–50 (U = 321.000, p = .050), 50–60 (U = 305.000, p < .050) and 60–70 (U = 282.000, p < .050).

**Conclusions:** PCI showed a more precise typification about how men of all ages understand the characteristics of cluttering. It is worth mentioning that men in nearby ages did not have substantial differences which indicates that they conceive cluttering in a similar way. In conclusion, older men have a totally different perception about this fluency disorder compared to men of younger ages and this can be useful evidence data during diagnosis [1–9].

**References**

1. Fountoulakis KN, Iacovides K, Kleanthous S, Samoilis S, S Kaprinis G, Sitzoglou K, Bech P: Reliability, validity and psychometric properties of the Greek translation of the Center for Epidemiological Studies-Depression (CES-D) Scale. BMC Psych 2001, 1(2): 3
2. Tafiadis D, Chronopoulos S K, Kosma E I et al. Using Receiver Operating Characteristic Curve to Define the Cutoff Points of Voice Handicap Index Applied to Young Adult Male Smokers. Journal of Voice. 2018;32(4):443–448. 10.1016/j.jvoice.2017.06.007
3. Tafiadis D, Kosma E I, Chronopoulos S K et al. Voice Handicap Index and Interpretation of the Cutoff Points Using Receiver Operating Characteristic Curve as Screening for Young Adult Female Smokers. Journal of Voice. 2018;32(1):64–69. 10.1016/j.jvoice.2017.03.009
4. Tafiadis D, Chronopoulos S, Siafaka V et al. Comparison of Voice Handicap Index Scores Between Female Students of Speech Therapy and Other Health Professions. Journal of Voice. 2017;31(5):583–588. 10.1016/j.jvoice.2017.01.013
5. Tafiadis D, Kosma E, Chronopoulos S et al. Acoustic and Perceived Measurements Certifying Tango as Voice Treatment Method. Journal of Voice. 2018;32(2): 256.e13–256.e24. 10.1016/j.jvoice.2017.05.016
6. Voniati L. School Based Speech and Language Therapists’ Perceptions of MLU-w and Its Use. Science Journal of Education. 2015; 3(6):119–125. 10.11648/j.sjedu.20150306.11
7. Voniati L. Mean Length of Utterance in CYG-speaking children. Journal of Greek Linguistics. 2016; 16(1): 117–140.
8. Voniati L. Christopoulou M. Developing an Augmentative and Alternative Communication System for a Child with Autism Spectrum Disorder: A Case Study, American Journal of Health Research. 2017; 1(2): 58–62. 10.11648/j.ijcsd.20170102.15
9. Voniati L. Charalambous, I. Rett syndrome: Clinical recognition, communication skills and therapeutic intervention, Archives of Hellenic Medicine. 2018; 35(2): 188–197.

**Ethics Approval:** The study was approved by the Ethical Committee of Department of Speech Language Therapy (School of Health and Welfare Professions) TEI of Epirus

**Consent to publish:** Informed consent to publish has been obtained from each participant.

## A36 Perceived levels of fluency, statistically analysed for typical female adults using predictive data processing of cluttering inventory: a pilot study in Greek population

### Maria Cherkeletzi^1^, Eleni Kapeta^1^, Soultana Soglemezi^1^, Evangelia I. Kosma^2,3^, Spyridon K. Chronopoulos^4,5^, Dionysios Tafiadis^1^

#### ^1^Department of Speech & Language Therapy, Technological Educational Institute of Epirus, Ioannina, Greece; ^2^Faculty of Medicine, School of Health Sciences, University of Thessaly, Biopolis, Larissa, Greece; ^3^Psychologist, Private Practice, Mihail Aggelou 18, Ioannina, Greece; ^4^Department of Informatics and Telecommunications Engineering, University of Western Macedonia, Kozani, Greece; ^5^Department of Computer Engineering, Technological Educational Institute of Epirus, Arta, Greece

##### **Correspondence:** Dionysios Tafiadis - d.tafiadis@ioa.teiep.gr; tafiadis@gmail.com

*Annals of General Psychiatry* 2018, **17(Suppl 1):**A36

**Background:** Fluency disorders affects 1% of the population and over 3 million people in the United States. Cluttering is a disorder characterized by a rapid rate of speech that may appear as non-proper erratic pronunciation. Many guidelines were suggested for the evaluation of cluttering to the literature and one diagnostic procedure is the use of self-perceived questionnaires. A self-reported questionnaire for adults cluttering is the “Predictive Cluttering Inventory’’. The aim of this study was to obtain evident based information in typical female population about cluttering using PCI.

**Materials and methods:** The Greek version of PCI was fulfilled by 181 typical women. Participants that had a history of medical conditions or factors that affected the administration procedures were excluded. The sample had a mean age of M = 49.09 (SD = 18.746) and was split into 6 age subgroups (18–30 to 70+).

**Results:** The study has shown statistically significant differences regarding all age groups for PCI’s total score [Η(5) = 42.672, p = .000]. Similarly, statistically significant differences were calculated between 18 and 30 age subgroup and all the other age spectrum: 30–40 (U = 228.000, p = .001), 40–50 (U = 301.000, p < .050), 50–60 (U = 142.000, p = .000), 60–70 (U = 132.000, p = .000) and 70 plus (U = 106.000, p = .000). Differences were noticed also between some age groups.

**Conclusions:** Young female population differs to a great extent from the middle-aged and elderly women relevant to understanding cluttering and its characteristics as in other disorders [1–9]. In conclusion, women from and after the middle 30 s seem to have a similar way to face cluttering and its characteristics.

**References**

1. Fountoulakis KN, Iacovides K, Kleanthous S, Samoilis S, S Kaprinis G, Sitzoglou K, Bech P: Reliability, validity and psychometric properties of the Greek translation of the Center for Epidemiological Studies-Depression (CES-D) Scale. BMC Psych 2001, 1(2): 3
2. Tafiadis D, Chronopoulos S K, Kosma E I et al. Using Receiver Operating Characteristic Curve to Define the Cutoff Points of Voice Handicap Index Applied to Young Adult Male Smokers. Journal of Voice. 2018;32(4):443–448. 10.1016/j.jvoice.2017.06.007
3. Tafiadis D, Kosma E I, Chronopoulos S K et al. Voice Handicap Index and Interpretation of the Cutoff Points Using Receiver Operating Characteristic Curve as Screening for Young Adult Female Smokers. Journal of Voice. 2018;32(1):64–69. 10.1016/j.jvoice.2017.03.009
4. Tafiadis D, Chronopoulos S, Siafaka V et al. Comparison of Voice Handicap Index Scores Between Female Students of Speech Therapy and Other Health Professions. Journal of Voice. 2017;31(5):583–588. 10.1016/j.jvoice.2017.01.013
5. Tafiadis D, Kosma E, Chronopoulos S et al. Acoustic and Perceived Measurements Certifying Tango as Voice Treatment Method. Journal of Voice. 2018;32(2): 256.e13–256.e24. 10.1016/j.jvoice.2017.05.016
6. Voniati L. School Based Speech and Language Therapists’ Perceptions of MLU-w and Its Use. Science Journal of Education. 2015; 3(6):119–125. 10.11648/j.sjedu.20150306.11
7. Voniati L. Mean Length of Utterance in CYG-speaking children. Journal of Greek Linguistics. 2016; 16(1): 117–140.
8. Voniati L. Christopoulou M. Developing an Augmentative and Alternative Communication System for a Child with Autism Spectrum Disorder: A Case Study, American Journal of Health Research. 2017; 1(2): 58–62. 10.11648/j.ijcsd.20170102.15
9. Voniati L. Charalambous, I. Rett syndrome: Clinical recognition, communication skills and therapeutic intervention, Archives of Hellenic Medicine. 2018; 35(2): 188–197.

## **Ethics Approval:** The study was approved by the Ethical Committee of Department of Speech Language Therapy (School of Health and Welfare Professions) TEI of Epirus

**Consent to publish:** Informed consent to publish has been obtained from each participant.

## A37 Validation data processing of premonitory awareness in stuttering scale: a pilot study in typical Greek population

### Maria-Eleni Kourmouta^1^, Fotini Mytilineou^1^, Christos Mokos^1^, Eirini Ntintila^1^, Dimitra Pantechi^1^, Sevasti Sakoglou^1^, Artemisia Halkia^1^, Evangelia I. Kosma^2,3^, Spyridon K. Chronopoulos^4,5^, Dionysios Tafiadis^1^

#### ^1^Department of Speech & Language Therapy, Technological Educational Institute of Epirus, Ioannina, Greece; ^2^Faculty of Medicine, School of Health Sciences, University of Thessaly, Biopolis, Larissa, Greece; ^3^Psychologist, Private Practice, Mihail Aggelou 18, Ioannina, Greece; ^4^Department of Informatics and Telecommunications Engineering, University of Western Macedonia, Kozani, Greece; ^5^Department of Computer Engineering, Technological Educational Institute of Epirus, Arta, Greece

##### **Correspondence:** Dionysios Tafiadis - d.tafiadis@ioa.teiep.gr; tafiadis@gmail.com

*Annals of General Psychiatry* 2018, **17(Suppl 1):**A37

**Background:** Anticipation of stuttering and its symptoms is a frequent but inadequately measured phenomenon of high importance in clinical evidence based practice. It is common in assessment procedure of stuttering—especially in kids—to use self-perceived questionnaires as in other disorders [1–9]. In the literature only one questionnaire is referred to adults’ self-awareness of stuttering, that is the Premonitory Awareness in Stuttering Scale (PAiS). The purpose of this study was the validation of PAiS questionnaire for typical Greek population.

**Materials and methods:** A total of 862 adults (440 females–422 males) aged 18 to 85 years old (M = 46.67 SD = 18.69) were recruited. They were all administrated PAiS questionnaire (a 12-items questionnaire) in order to self-evaluate their fluency of speech. The questionnaire was translated and adapted to Greek language according to minimal translation criteria.

**Results:** Data analysis showed no statistical significant differences for PAiS total score between male and female participants (U = 89296.000, NS) and all age subgroups [H(5) = 7.792, NS]. Significant differences of Medians were observed between male and female in questions Q1: (U = 86753.000, p < .050), Q2: (87506.000, p < .050) and Q4: (U = 87071.000, p < .050). PAis questionnaire had high internal consistency (Reliability Coefficients 12 items Alpha = .949).

**Conclusions:** PAiS exhibits the potentiality of quantifying the perceived level of a person’s fluency abilities. PAiS can be used across all the adult lifespan as a premonitory awareness tool and give adequate data for evidence clinical practice. Furthermore, Greek version of PAiS sounds appropriate for use and it is suggested to be standardized for Greek reality.

**References**

1. Tafiadis D, Kosma E I, Chronopoulos S K et al. Voice Handicap Index and Interpretation of the Cutoff Points Using Receiver Operating Characteristic Curve as Screening for Young Adult Female Smokers. Journal of Voice. 2018;32(1):64–69. 10.1016/j.jvoice.2017.03.009
2. Tafiadis D, Chronopoulos S K, Kosma E I et al. Using Receiver Operating Characteristic Curve to Define the Cutoff Points of Voice Handicap Index Applied to Young Adult Male Smokers. Journal of Voice. 2018;32(4):443–448. 10.1016/j.jvoice.2017.06.007
3. Tafiadis D, Helidoni M, Chronopoulos S et al. Preliminary Receiver Operating Characteristic Analysis on Voice Handicap Index of Laryngeal Inflammation in Greek Patients. International Journal of Otolaryngology and Head & Neck Surgery. 2018;07(03):115–131. 10.4236/ijohns.2018.73014
4. Tafiadis D, Kosma E, Chronopoulos S, Voniati L, Ziavra N. A Preliminary Receiver Operating Characteristic Analysis on Voice Handicap Index Results of the Greek Voice-Disordered Patients. International Journal of Otolaryngology and Head & Neck Surgery. 2018;07(03):98–114. 10.4236/ijohns.2018.73013
5. Chronopoulos S, Kosma E, Tafiadis D et al. Reduced Ecological Footprints of Modern Facilities Introducing the Implementation of Advanced Wireless Technologies, and Human Resources’ Benefits. Communications and Network. 2018;10(01):11–29. 10.4236/cn.2018.101002
6. Voniati L. School Based Speech and Language Therapists’ Perceptions of MLU-w and Its Use. Science Journal of Education. 2015; 3(6):119–125. 10.11648/j.sjedu.20150306.11
7. Voniati L. Mean Length of Utterance in CYG-speaking children. Journal of Greek Linguistics. 2016; 16(1): 117–140.
8. Voniati L. Christopoulou M. Developing an Augmentative and Alternative Communication System for a Child with Autism Spectrum Disorder: A Case Study, American Journal of Health Research. 2017; 1(2): 58–62. 10.11648/j.ijcsd.20170102.15
9. Voniati L. Charalambous, I. Rett syndrome: Clinical recognition, communication skills and therapeutic intervention, Archives of Hellenic Medicine. 2018; 35(2): 188–197.

**Ethics Approval:** The study was approved by the Ethical Committee of Department of Speech Language Therapy (School of Health and Welfare Professions) TEI of Epirus

**Consent to publish:** Informed consent to publish has been obtained from each participant.

## A38 Determining through data analysis the Mean Length of Utterance in Morphemes (MLUm) and Mean Length of Utterance in Words (MLUw) in Cypriot Greek speaking children

### Louiza Voniati^1^, Myria Adamou^1^, Evangelia I. Kosma^2,3^, Spyridon K. Chronopoulos^4,5^, Dionysios Tafiadis^6^

#### ^1^Department of Speech & Language Therapy, European University of Cyprus, Nicosia, Cyprus; ^2^Faculty of Medicine, School of Health Sciences, University of Thessaly, Biopolis, Larissa, Greece; ^3^Psychologist, Private Practice, Mihail Aggelou 18, Ioannina, Greece; ^4^Department of Informatics and Telecommunications Engineering, University of Western Macedonia, Kozani, Greece; ^5^Department of Computer Engineering, Technological Educational Institute of Epirus, Arta, Greece; ^6^Department of Speech & Language Therapy, Technological Educational Institute of Epirus, Ioannina, Greece

##### **Correspondence:** Dionysios Tafiadis - d.tafiadis@ioa.teiep.gr; tafiadis@gmail.com

*Annals of General Psychiatry* 2018, **17(Suppl 1):**A38

**Background:** The collection and analysis of a language sample is of particular research and clinical interest due to its high validity [1–5]. The most widely used language indicator is the Mean Length of Utterance (MLU). MLU is usually counted in words (MLU-w) or morphemes (MLU-m). A brief review of the literature revealed a lack of research evidence which is whether MLU-w or MLU-m would be more suitable for use in children speaking the Cypriot Greek dialect [6–7]. The aim of this study was to compare MLU-w and MLU-m with children Cypriot Greek dialect of typical language development aged from 36 to 48 months.

**Materials and methods:** This study was based on existing data, from the Early Childhood Re- search Laboratory Centre of the University of Cyprus database. Thirty children aged from 36 to 48 months, of typical linguistic development, participated in this study. In 12 months duration, the children underwent three re-evaluations every 4 months and in particular 36, 40, 44 and 48 months. The children who had language or any other disorder and did not come from a mono- lingual Cypriot Greek dialect family were excluded from this study.

**Results:** According to the results at each specified age point (36, 40, 44 & 48 months), the children showed very high positive correlation to the association of MLU-w and MLU-m.

**Conclusions:** MLU-w could be presented as an assessment tool for Cypriot Greek speakers and will contribute to a valid diagnosis [8–9]. However, it is noteworthy that the findings of this study reflect an average beneficial level. Therefore, an individual’s linguistic development may deviate from the standards described in this research.

**References**

1. Tafiadis D, Kosma EI, Chronopoulos SK et al. Voice Handicap Index and Interpretation of the Cutoff Points Using Receiver Operating Characteristic Curve as Screening for Young Adult Female Smokers. Journal of Voice. 2018;32(1):64–69. 10.1016/j.jvoice.2017.03.009
2. Tafiadis D, Chronopoulos S K, Kosma E I et al. Using Receiver Operating Characteristic Curve to Define the Cutoff Points of Voice Handicap Index Applied to Young Adult Male Smokers. Journal of Voice. 2018;32(4):443–448. 10.1016/j.jvoice.2017.06.007
3. Tafiadis D, Helidoni M, Chronopoulos S et al. Preliminary Receiver Operating Characteristic Analysis on Voice Handicap Index of Laryngeal Inflammation in Greek Patients. International Journal of Otolaryngology and Head & Neck Surgery. 2018;07(03):115–131. 10.4236/ijohns.2018.73014
4. Tafiadis D, Kosma E, Chronopoulos S, Voniati L, Ziavra N. A Preliminary Receiver Operating Characteristic Analysis on Voice Handicap Index Results of the Greek Voice-Disordered Patients. International Journal of Otolaryngology and Head & Neck Surgery. 2018;07(03):98–114. 10.4236/ijohns.2018.73013
5. Chronopoulos S, Kosma E, Tafiadis D et al. Reduced Ecological Footprints of Modern Facilities Introducing the Implementation of Advanced Wireless Technologies, and Human Resources’ Benefits. Communications and Network. 2018;10(01):11–29. 10.4236/cn.2018.101002
6. Voniati L. School Based Speech and Language Therapists’ Perceptions of MLU-w and Its Use. Science Journal of Education. 2015; 3(6):119–125. 10.11648/j.sjedu.20150306.11
7. Voniati L. Mean Length of Utterance in CYG-speaking children. Journal of Greek Linguistics. 2016; 16(1): 117–140.
8. Voniati L. Christopoulou M. Developing an Augmentative and Alternative Communication System for a Child with Autism Spectrum Disorder: A Case Study, American Journal of Health Research. 2017; 1(2): 58–62. 10.11648/j.ijcsd.20170102.15
9. Voniati L. Charalambous, I. Rett syndrome: Clinical recognition, communication skills and therapeutic intervention, Archives of Hellenic Medicine. 2018; 35(2): 188–197.

**Ethics Approval:** The study was approved by the Ethical Committee of Department of Speech Language Therapy (School of Health and Welfare Professions) TEI of Epirus.

**Consent to publish:** Informed consent to publish has been obtained from each participant.

## A39 Derivative and descriptive data analysis on pragmatics of pre-primary cypriot greek speaking children

### Louiza Voniati^1^, Emily Argyrou^1^, Maria Mita^1^, Evangelia I. Kosma^2,3^, Spyridon K. Chronopoulos^4,5^, Dionysios Tafiadis^6^

#### ^1^Department of Speech & Language Therapy, European University of Cyprus, Nicosia, Cyprus; ^2^Faculty of Medicine, School of Health Sciences, University of Thessaly, Biopolis, Larissa, Greece; ^3^Psychologist, Private Practice, Mihail Aggelou 18, Ioannina, Greece; ^4^Department of Informatics and Telecommunications Engineering, University of Western Macedonia, Kozani, Greece; ^5^Department of Computer Engineering, Technological Educational Institute of Epirus, Arta, Greece; ^6^Department of Speech & Language Therapy, Technological Educational Institute of Epirus, Ioannina, Greece

##### Dionysios TafiadisDionysios Tafiadis - d.tafiadis@ioa.teiep.gr; tafiadis@gmail.com

*Annals of General Psychiatry* 2018, **17(Suppl 1):**A39

**Background:** The factual study examines the use of language in a social context. The purpose of the research was to examine the use of language in 4-year-old children of typical language development, speaking the Cypriot dialect.

**Materials and methods:** A total of 20 children (10 boys and 10 girls), who were Cypriot dialect speakers with typical language development, participated in the study. The language sample was collected using free play and free conversation and was recorded with a digital recorder in the home of each child. The language sample size consisted of 50 enunciations from each child, and the transcript of the language sample was written by hand and it was based on the International Phonetic Alphabet. The results were analyzed using descriptive statistics.

**Results:** The results of the study showed that most language usage categories (Commentary, Regulation, Protest or Rejection, Emotion, Routine, Report or Inform, Performance and Narration) appeared in the language sample of both boys and girls. Four of the eight categories (Report or Inform, Commentary, Response and Refusal) were in most cases observed in both genders. Notably, there was a statistical significance in the gender discrimination category (P < 0.04).

**Conclusions:** The results of this study can be used in a preliminary basis for comparisons relevant to populations who are likely to suffer from speech and communication disorders [1–6].

**References**

1. Voniati L. School Based Speech and Language Therapists’ Perceptions of MLU-w and Its Use. Science Journal of Education. 2015; 3(6):119–125. 10.11648/j.sjedu.20150306.11
2. Voniati L. Mean Length of Utterance in CYG-speaking children. Journal of Greek Linguistics. 2016; 16(1): 117–140.
3. Voniati L. Christopoulou M. Developing an Augmentative and Alternative Communication System for a Child with Autism Spectrum Disorder: A Case Study, American Journal of Health Research. 2017; 1(2): 58–62. 10.11648/j.ijcsd.20170102.15
4. Voniati L. Charalambous, I. Rett syndrome: Clinical recognition, communication skills and therapeutic intervention, Archives of Hellenic Medicine. 2018; 35(2): 188–197.
5. Voniati L. (2016) The Effect of Gender on Transactive Dialogues In Peer Collaboration. IOSR Journal οf Humanities And Social Science. 2016; 21(3): 22–27.
6. Voniati L. Extended day implementation in special education: Leadership characteristics - a case study, IOSR Journal of Research & Method in Education. 2017; 7(3): 01–06.

**Ethics Approval:** The study was approved by the Ethical Committee of Department of Speech Language Therapy (School of Health and Welfare Professions) TEI of Epirus.

**Consent to publish:** Informed consent to publish has been obtained from each participant.

## A40 The contribution of the communication partner in language sample analysis

### Louiza Voniati^1^, Despo Leontiou^1^, Patricia Kyriacou^1^, Evangelia I. Kosma^2,3^, Spyridon K. Chronopoulos^4,5^, Dionysios Tafiadis^6^

#### ^1^Department of Speech & Language Therapy, European University of Cyprus, Nicosia, Cyprus; ^2^Faculty of Medicine, School of Health Sciences, University of Thessaly, Biopolis, Larissa, Greece; ^3^Psychologist, Private Practice, Mihail Aggelou 18, Ioannina, Greece; ^4^Department of Informatics and Telecommunications Engineering, University of Western Macedonia, Kozani, Greece; ^5^Department of Computer Engineering, Technological Educational Institute of Epirus, Arta, Greece; ^6^Department of Speech & Language Therapy, Technological Educational Institute of Epirus, Ioannina, Greece

##### **Correspondence:** Dionysios Tafiadis - d.tafiadis@ioa.teiep.gr; tafiadis@gmail.com

*Annals of General Psychiatry* 2018, **17(Suppl 1):**A40

**Background:** Language is a way of expression which is inherent in all people. Each person is born with the ability to develop language irrespective of nationality or language. However, some children may have language disorder or they may not have typical language development [1–4]. In order to diagnose a language disorder, clinicians use formal or informal assessments. Language sample collection and analysis is an informal assessment that provides data from spontaneous speech productions on real communication environments. The main objective of the study was to find the influence of the conversational partner in Cypriot-Greek speaking children with typical language development.

**Materials and methods:** The research was conducted at the Speech, Language and Hearing Clinic of the European University Cyprus. The sample consisted of 20 3-year-old Cypriot-Greek speaking children 3-years-old with typical language development. Each child’s voice was recorded inside clinic at a dyadic interaction with one of his/her parent and afterwards at a dyadic interaction with one of the researchers. Furthermore, the Number of Different Words (NDW) and the Mean Length Utterance-words (MLU-w) of the child with both communicational partners were analyzed and finally a statistical analysis and comparison of data was conducted.

**Results:** The results showed that the MLU-w did not differ significantly. However, the NDW was significantly higher when a child was interacting with a parent.

**Conclusions:** Findings of the research give important information on the influence of the communication partner in children’s speech and could help clinicians towards choosing the right person which will interact with a child when collecting a language sample [5–9].

**References**

1. Voniati L. School Based Speech and Language Therapists’ Perceptions of MLU-w and Its Use. Science Journal of Education. 2015; 3(6):119–125. 10.11648/j.sjedu.20150306.11
2. Voniati L. Mean Length of Utterance in CYG-speaking children. Journal of Greek Linguistics. 2016; 16(1): 117–140.
3. Voniati L. Christopoulou M. Developing an Augmentative and Alternative Communication System for a Child with Autism Spectrum Disorder: A Case Study, American Journal of Health Research. 2017; 1(2): 58–62. 10.11648/j.ijcsd.20170102.15
4. Voniati L. Charalambous, I. Rett syndrome: Clinical recognition, communication skills and therapeutic intervention, Archives of Hellenic Medicine. 2018; 35(2): 188–197.
5. Voniati L. (2016) The Effect of Gender on Transactive Dialogues In Peer Collaboration. IOSR Journal οf Humanities and Social Science. 2016; 21(3): 22–27.
6. Voniati L. Extended day implementation in special education: Leadership characteristics - a case study, IOSR Journal of Research & Method in Education. 2017; 7(3): 01–06.
7. Tafiadis D, Chronopoulos S K, Kosma E I et al. Using Receiver Operating Characteristic Curve to Define the Cutoff Points of Voice Handicap Index Applied to Young Adult Male Smokers. Journal of Voice. 2018;32(4):443–448. 10.1016/j.jvoice.2017.06.007
8. Tafiadis D, Kosma E, Chronopoulos S et al. Acoustic and Perceived Measurements Certifying Tango as Voice Treatment Method. Journal of Voice. 2018;32(2):256.e13–256.e24. 10.1016/j.jvoice.2017.05.016
9. Tafiadis D, Chronopoulos S, Siafaka V et al. Comparison of Voice Handicap Index Scores Between Female Students of Speech Therapy and Other Health Professions. Journal of Voice. 2017;31(5):583–588. 10.1016/j.jvoice.2017.01.013

**Ethics Approval:** The study was approved by the Ethical Committee of Department of Speech Language Therapy (School of Health and Welfare Professions) TEI of Epirus.

**Consent to publish:** Informed consent to publish has been obtained from each participant.

## A41 Analysing the data acquisition of communication skills of children with autism spectrum disorders: the parents’ opinions

### Louiza Voniati^1^, Maria Pasiourtidou^1^, Andrea Lemeshi^1^, Evangelia I. Kosma^2,3^, Spyridon K. Chronopoulos^4,5^, Dionysios Tafiadis^6^

#### ^1^Department of Speech & Language Therapy, European University of Cyprus, Nicosia, Cyprus; ^2^Faculty of Medicine, School of Health Sciences, University of Thessaly, Biopolis, Larissa, Greece; ^3^Psychologist, Private Practice, Mihail Aggelou 18, Ioannina, Greece; ^4^Department of Informatics and Telecommunications Engineering, University of Western Macedonia, Kozani, Greece; ^5^Department of Computer Engineering, Technological Educational Institute of Epirus, Arta, Greece; ^6^Department of Speech & Language Therapy, Technological Educational Institute of Epirus, Ioannina, Greece

##### **Correspondence:** Dionysios Tafiadis - d.tafiadis@ioa.teiep.gr; tafiadis@gmail.com

*Annals of General Psychiatry* 2018, **17(Suppl 1):**A41

**Background:** The current research studied in depth the actual views of the parents of Greek Cypriot children with autistic spectrum disorder regarding the communication skills of their children at pre-school and primary school age.

**Materials and methods:** The data were collected through qualitative research. The chosen method was a structured interview which consisted of nineteen open questions. The interview was recorded using an electronic tape recorder in order to get a precise data recording. The collected data were recorded on tables which were created according to the posed questions during interview.

**Results:** The results of the study exhibited that parents of children with autistic spectrum disorder were well informed about the therapeutic/educational programs that a child with the specific disorder had to attend. In addition, the research showed that the parents were well aware of the various ways which their child uses to communicate in different environments both in and out of their home. Furthermore, through this study it was revealed that children with autistic spectrum disorder were not approached in the best appropriate way by parents, siblings and familiar people and this was due to lack of knowledge regarding the proper ways of approaching and communicating with children with autistic spectrum disorder.

**Conclusions:** The limitations of this study were noted and suggestions were given regarding possible future research on children with autistic spectrum disorder in order to help not only the parents but also therapists who come into contact with this specific disorder [1–6].

**References**

1. Voniati L. School Based Speech and Language Therapists’ Perceptions of MLU-w and Its Use. Science Journal of Education. 2015; 3(6):119–125. 10.11648/j.sjedu.20150306.11
2. Voniati L. Mean Length of Utterance in CYG-speaking children. Journal of Greek Linguistics. 2016; 16(1): 117–140.
3. Voniati L. Christopoulou M. Developing an Augmentative and Alternative Communication System for a Child with Autism Spectrum Disorder: A Case Study, American Journal of Health Research. 2017; 1(2): 58–62. 10.11648/j.ijcsd.20170102.15
4. Voniati L. Charalambous, I. Rett syndrome: Clinical recognition, communication skills and therapeutic intervention, Archives of Hellenic Medicine. 2018; 35(2): 188–197.
5. Voniati L. (2016) The Effect of Gender on Transactive Dialogues In Peer Collaboration. IOSR Journal οf Humanities and Social Science. 2016; 21(3): 22–27.
6. Voniati L. Extended day implementation in special education: Leadership characteristics - a case study, IOSR Journal of Research & Method in Education. 2017; 7(3): 01–06.

**Ethics Approval:** The study was approved by the Ethical Committee of Department of Speech Language Therapy (School of Health and Welfare Professions) TEI of Epirus.

**Consent to publish:** Informed consent to publish has been obtained from each participant.

## A42 Validating the autism spectrum screening questionnaire via data analysis: a pilot study in a typical Greek population

### Nikoleta Alexopoulou^1^, Evangelia I. Kosma^2,3^, Spyridon K. Chronopoulos^4,5^, Dionysios Tafiadis^6^

#### ^1^Department of Humanities and Social Sciences, University of Strathclyde, Glasgow, United Kingdom; ^2^Faculty of Medicine, School of Health Sciences, University of Thessaly, Biopolis, Larissa, Greece; ^3^Psychologist, Private Practice, Mihail Aggelou 18, Ioannina, Greece; ^4^Department of Informatics and Telecommunications Engineering, University of Western Macedonia, Kozani, Greece; ^5^Department of Computer Engineering, Technological Educational Institute of Epirus, Arta, Greece; ^6^Department of Speech & Language Therapy, Technological Educational Institute of Epirus, Ioannina, Greece

##### **Correspondence:** Dionysios Tafiadis - d.tafiadis@ioa.teiep.gr; tafiadis@gmail.com

*Annals of General Psychiatry* 2018, **17(Suppl 1):**A42

**Background:** Quite a few questionnaires have been created for the detection of autistic characteristics of average population. One of them is the Autism Spectrum Screening Questionnaire (ASSQ) [1]. It is consisted of 27 questions which detect impairments in socialization, communication and unfamiliar behaviors. ASSQ has been satisfactorily validated and used in clinical practice to general population as well as to clinical samples [1–2]. Nevertheless, it has not been translated nor validated in Greek language. Also, few researches exist that evaluate the gender and age differences depending on the given answers [3–4]. The purpose of this study was the translation of ASSQ questionnaire and validation in Greek-nonprofessional sample-typically developed parents.

**Materials and methods:** The ASSQ questionnaire was translated in Greek language and administered to a population of 361 typically developed adults. All these adults spoke Greek as their first language (137 men and 224 women). Their age varied from 18 to 60 years old.

**Results:** The ASSQ screening questionnaire was filled in by all the participants. The reliability of the AQ questionnaire was satisfactory in its Greek translation with a Cronbach Alpha being equal to 0.872. The internal coherence was also strong. In general, males’ scores were higher than females’ scores based on the analysis of the ASSQ questionnaire. Results showed statistically major differences at four answers between men and women, but not in the total score. Also, when age groups were compared, differences were notable in eight answers, which did not affect the total score as well.

**Conclusions:** ASSQ constitutes a screening questionnaire that is well validated in other languages except Greek. It is used as a diagnostic tool and can detect High Functioning Autism and AS. Even though it was translated and given successfully to a Greek population sample, more researches need to be conducted. Gender and age differences did not play a notable role to the total score, but there where remarkable differences to each answer [3–7]. Therefore, further studies on larger samples are needed to confirm the observed gender and age differences [3–7].

**References**

1. Ehlers S Gillberg, C Wing, L. A screening questionnaire for Asperger syndrome and other high-functioning autism spectrum disorders in school age children. Journal of Autism and Developmental Disorders. 1999; 29(2): 129–141.
2. Posserud M B, Lundervold A J, Gillberg C. Validation of the autism spectrum screening questionnaire in a total population sample. Journal of Autism and Developmental Disorders. 2009; 39(1): 126–134.
3. Tafiadis D, Kosma E I, Chronopoulos S K et al. Voice Handicap Index and Interpretation of the Cutoff Points Using Receiver Operating Characteristic Curve as Screening for Young Adult Female Smokers. Journal of Voice. 2018;32(1):64–69. 10.1016/j.jvoice.2017.03.009
4. Tafiadis D, Chronopoulos S K, Kosma E I et al. Using Receiver Operating Characteristic Curve to Define the Cutoff Points of Voice Handicap Index Applied to Young Adult Male Smokers. Journal of Voice. 2018;32(4):443–448. 10.1016/j.jvoice.2017.06.007
5. Tafiadis D, Helidoni M, Chronopoulos S et al. Preliminary Receiver Operating Characteristic Analysis on Voice Handicap Index of Laryngeal Inflammation in Greek Patients. International Journal of Otolaryngology and Head & Neck Surgery. 2018;07(03):115–131. 10.4236/ijohns.2018.73014
6. Tafiadis D, Kosma E, Chronopoulos S, Voniati L, Ziavra N. A Preliminary Receiver Operating Characteristic Analysis on Voice Handicap Index Results of the Greek Voice-Disordered Patients. International Journal of Otolaryngology and Head & Neck Surgery. 2018;07(03):98–114. 10.4236/ijohns.2018.73013
7. Chronopoulos S, Kosma E, Tafiadis D et al. Reduced Ecological Footprints of Modern Facilities Introducing the Implementation of Advanced Wireless Technologies, and Human Resources’ Benefits. Communications and Network. 2018;10(01):11–29. 10.4236/cn.2018.101002

**Ethics Approval:** The study was approved by the Ethical Committee of Department of Speech Language Therapy (School of Health and Welfare Professions) TEI of Epirus.

**Consent to publish:** Informed consent to publish has been obtained from each participant.

## A43 Data analysis for the validation of the autism-spectrum quotient: a pilot study in a typical Greek population

### Nikoleta Alexopoulou^1^, Evangelia I. Kosma^2,3^, Spyridon K. Chronopoulos^4,5^, Dionysios Tafiadis^6^

#### ^1^Department of Humanities and Social Sciences, University of Strathclyde, Glasgow, United Kingdom; ^2^Faculty of Medicine, School of Health Sciences, University of Thessaly, Biopolis, Larissa, Greece; ^3^Psychologist, Private Practice, Mihail Aggelou 18, Ioannina, Greece; ^4^Department of Informatics and Telecommunications Engineering, University of Western Macedonia, Kozani, Greece; ^5^Department of Computer Engineering, Technological Educational Institute of Epirus, Arta, Greece; ^6^Department of Speech & Language Therapy, Technological Educational Institute of Epirus, Ioannina, Greece

##### **Correspondence:** Dionysios Tafiadis - d.tafiadis@ioa.teiep.gr; tafiadis@gmail.com

*Annals of General Psychiatry* 2018, **17(Suppl 1):**A43

**Background:** Individuals, in the spectrum of autism, present difficulties in “empathising” and show higher results in “systemising” [1]. In general, women empathise more than men and in turn males systemise more than females [1–7]. Autism-Spectrum Quotient (AQ) is a 50-item self-administered questionnaire which was created to measure the level of autistic traits to adults with normal intelligence [8]. This questionnaire is consisted of questions based on measuring the main impairments confronted by individuals with ASD. The questions are also based on the ability of males and females to empathise and systemize. The aim of this study was the translation of the AQ questionnaire in Greek language and its validation in Greek non-professional population-typically developed parents.

**Materials and methods:** The AQ questionnaire was translated in Greek language and administered to a population of 325 typically developed adults. All of them spoke Greek as their first language (150 men and 175 women). Their age varied from 19 years old to 75+ years.

**Results:** All the participants completed the AQ questionnaire. The reliability of the AQ questionnaire was satisfactory in its Greek translation with Cronbach Alpha being equal to 0.707. The internal coherence was also strong. The analysis of the results showed that differences in the answers existed between males and females to specific questions but not in the total score. It is notable that males present a higher score to their answers than women do. Also, analysis showed remarkable differences to the answers in specific questions between the age groups as well as to the total score.

**Conclusions:** AQ is a questionnaire which can be used as a tool for the detection of autism spectrum disorders. It can provide important information about the social interaction and communication deficits faced by an individual with an average IQ level. The Greek translation of the questionnaire was rated as satisfactory. Nevertheless, further research would be useful for the validation of the AQ in Greek language and for the detection of the age and gender differences from the answers.

**References**

1. Baron-Cohen S. The extreme male brain theory of autism. Trends on Cognitive Science 2002; 6(6): 248–254.
2. Baron-Cohen S, Wheelwright S, Skinner R, Martin J, Clubley E. The autism-spectrum quo- tient (AQ): Evidence from asperger syndrome/high-functioning autism, males and females, scientists and mathematicians Journal of Autism and Developmental Disorders. 2001; 31(1): 5–17.
3. Tafiadis D, Kosma E I, Chronopoulos S K et al. Voice Handicap Index and Interpretation of the Cutoff Points Using Receiver Operating Characteristic Curve as Screening for Young Adult Female Smokers. Journal of Voice. 2018;32(1):64–69. 10.1016/j.jvoice.2017.03.009 
4. Tafiadis D, Chronopoulos S K, Kosma E I et al. Using Receiver Operating Characteristic Curve to Define the Cutoff Points of Voice Handicap Index Applied to Young Adult Male Smokers. Journal of Voice. 2018;32(4):443–448. 10.1016/j.jvoice.2017.06.007  
5. Tafiadis D, Helidoni M, Chronopoulos S et al. Preliminary Receiver Operating Characteristic Analysis on Voice Handicap Index of Laryngeal Inflammation in Greek Patients. International Journal of Otolaryngology and Head & Neck Surgery. 2018;07(03):115–131. 10.4236/ijohns.2018.73014
6. Tafiadis D, Kosma E, Chronopoulos S, Voniati L, Ziavra N. A Preliminary Receiver Operating Characteristic Analysis on Voice Handicap Index Results of the Greek Voice-Disordered Patients. International Journal of Otolaryngology and Head & Neck Surgery. 2018;07(03):98–114. 10.4236/ijohns.2018.73013
7. Chronopoulos S, Kosma E, Tafiadis D et al. Reduced Ecological Footprints of Modern Facilities Introducing the Implementation of Advanced Wireless Technologies, and Human Resources’ Benefits. Communications and Network. 2018;10(01):11–29. 10.4236/cn.2018.101002
8. Baron-Cohen S, R A Hoekstra, Knickmeyer R, Wheelwright S. The autism-spectrum quotient (AQ) - adolescent version. Journal of Autism and Developmental Disorders. 2006; 36(3): 343.

**Ethics Approval:** The study was approved by the Ethical Committee of Department of Speech Language Therapy (School of Health and Welfare Professions) TEI of Epirus.

**Consent to publish:** Informed consent to publish has been obtained from each participant.

## A44 Does premature menopause have an impact on cognitive function?

### Andreas Vardiampasis, Christina Gramandani

#### Mental Health Center, Rethymno, Crete, 74100, Greece

##### **Correspondence:** Andreas Vardiampasis - avardiampasis@gmail.com

*Annals of General Psychiatry* 2018, **17(Suppl 1):**A44

**Background:** The most important difference between the world today and 150 years ago is lifespan. The average life expectancy for women in Greece is 81.59 years old (y.o.), therefore, taken into consideration that the average age at menopause is 50 y.o., a woman spends a considerable time in climacteric. Positive correlations between estrogen levels and cognitive functions have been reported in several studies. This study aims to determine whether premature menopause affects women’s cognitive function.

**Materials and Methods:** A sample of 30 women, aged between 60 and 75 y.o., who have visited our Memory Clinic. Fifteen of them had premature menopause (≤ 40 y.o.) and the other 15 had menopause after 50 y.o. We included women with similar educational level (> 9 years of education), without major depression, chronic physical illness, alcohol and tobacco abuse, Mini-mental State Examination score > 25, and Montreal Cognitive Assessment (MoCA) score > 26. Clinical data was gathered using the Verbal Fluency Test (VFT) and the Trail Making Test (TMT).

**Results:** Women with premature menopause scored at VFT Phonemic ms = 34.67, at VFT Semantic ms = 38.67, at TMT-A ms = 39.47 and at TMT-Β ms = 102.4, whereas women with menopause ≥ 50 had 44.53, 44.6, 25.93, and 81.47 (t-value = 3.21, 3.58, 3.97, and 4.13) respectively.

**Conclusions:** Results show a statistical significant difference between the two subgroups. Given the fact that premature menopause is associated with a negative impact on cognitive function it is crucial to investigate if the possible benefits from a hormone replacement therapy counterbalance the potential risk.

## A45 Do children’s sleep disturbances affect the sexual quality of life of their parents?

### Andreas Vardiampasis, Christina Gramandani

#### Mental Health Center, Rethymno, Crete, 74100, Greece

##### **Correspondence:** Andreas Vardiampasis - avardiampasis@gmail.com

*Annals of General Psychiatry* 2018, **17(Suppl 1):**A45

**Background:** Parenthood is often accompanied with changes to the lifestyle, in general, and to the sexual life of the parents, in particular. In this study we explore whether parents’ sexual quality of life is affected due to children’s sleep disturbances.

**Materials and Methods:** A sample of 43 Greek couples with children aged between 3and 6 y.o. completed the Sleep Disturbances Scale for Children (SDSC), the Sexual Quality of Life Questionnaire Male & Female (SQOL-M & SQOL-F). The first measures the presence of sleep disturbances to children 3–16 y.o., the past 6 months. SQOL-M & SQOL-F evaluate the correlation between sexual dysfunction and the quality of life, at aspects such as, sexual satisfaction, psychosexual feelings and relationship satisfaction. Scales were completed by both parents. The parents were entered into the study if they had similar educational level (> 9 years of education), and if they were without clinical depression, chronic disease, and alcohol abuse. Sixteen parents with children with severe sleep disturbances (SDSC score > 58) were compared to 15 parents with children without sleep disturbances (SDSC score < 41).

**Results:** There was a significant difference to the sexual quality of life between the group of mothers with children having severe sleep disturbances compared to the women of the control group (t-value = 4.94 & p-value = 0.00005 for p < 05), with the first being seriously negatively affected. No significant difference has been found among the two groups of fathers (t-value = 0.572 & p-value = 0.28 for p < 05).

**Conclusions:** Mother’s sexual quality of life is shown to be more affected compared to the father’s sexual quality of life due to their children’s sleep disturbances. Do women carry an objectively greater burden related to their children’s up-bringing? Or, maybe, their cognitions, and, therefore the way they respond to these issues are responsible for this difference? In any case, sexuality and sexual life are fundamental aspects of a relationship. A good sexual quality of life is an important milestone in a healthy parents’ relationship and an overall optimal quality of life.

## A46 Sensorial stimulation in vegetative state patients: what kind of response?

### Irene Venturella^1,2^, Marina Fossati^3^, Francesca Fiorillo^3^, Michela Balconi^1,2^

#### ^1^Research Unit in Affective and Social Neuroscience, Catholic University of the Sacred Heart, Milan, Italy; ^2^Department of Psychology, Catholic University of the Sacred Heart, Milan, Italy; ^3^RSA “Foscolo”, Gruppo La Villa spa, Guanzate, Como, Italy

##### **Correspondence:** Irene Venturella - irene.venturella@unicatt.it

*Annals of General Psychiatry* 2018, **17(Suppl 1):**A46

**Background** Responsivity assessment and preserved cognitive abilities detection in vegetative state (VS) patients is usually observation-driven and based on overt responses that are often difficult to classify. The same is true for inferring subjective experiences like somatosensory percept and pain. Thus, electrophysiological covert measures could be considered a helpful tool to provide remarkable information on patients’ responsivity to environmental stimuli. Residual processing of somatosensory and nociceptive stimuli in VS patients in absence of behavioural evidences were investigated by observing cortical oscillations and physiological activation.

**Materials and Methods** A sample of 22 VS patients received a wrist hold and the application of an ice pack to induce somatosensory activations related to, respectively, pleasant and unpleasant stimulations. Cortical activity (measured by EEG) and physiological activation measures were acquired during the sensorial stimulation.

**Results** Autonomic indices showed greater skin conductance levels and greater heart rate in response to ice compared to the wrist hold. EEG measures highlighted higher frontal and parietal delta band power evoked by both pleasant and unpleasant stimulations. Specifically, unpleasant stimulation aroused greater delta parietal responses than the pleasant ones, while pleasant stimulations were associated to greater decrease of alpha activity in left frontal areas.

**Conclusions** These patterns of neurophysiological activity might be interpreted as preserved basic vigilance responses to environmental stimuli in this cohort of patients. The analysis of EEG profiles during somatic stimulations might suggest informative patterns of different oscillatory responses (different modulations of alpha and delta activity), giving insights on the opportunity to test basic responsivity in patients with disorders of consciousness.

**Ethics Approval:** This study was approved by the Ethics Committee of the Department of Psychology of the Catholic University of the Sacred Heart of Milan, Italy.

**Consent to publish:** Written informed consent for publication of their clinical details was obtained from the legal representatives of the patients.

## A47 Improvement in the behaviour of psychotic inpatients: are vitamin D levels connected?

### Theofanis Vorvolakos

#### Department of Medicine, School of Health Sciences, Democritus University of Thrace, Alexandroupolis, Greece

##### **Correspondence:** Theofanis Vorvolakos

*Annals of General Psychiatry* 2018, **17(Suppl 1):**A47

The role of Vitamin D and the other neurosteroid factors in psychosis is widely argued during the last years.

Two schizophrenic inpatients in our department, one newly diagnosed and one more chronic demonstrated severe behavioral symptoms especially agitation that made it difficult for the clinical staff to handle. During the routine checkup extremely low levels of Vitamin D ware revealed in both cases.

Patients were treated with Vitamin D, with a dose of around 4000 Units per day and until their Vitamin D levels in the blood reach a concentration within the normal limits. Their psychotic symptoms and their behavior showed significant improvement. The improvement was demonstrated in Positive and Negative Rating Scale (PANSS) and Health of the Nation Outcome Scales (HoNOS).

Despite the fact that there might be confounders and that this observation was made only in a couple of cases, we concluded that the role of Vitamin D levels in psychosis deserves more attention and needs further investigation.

After initial literature review that supported our observations we collaborated with the Molecular Biology and Genetics department of our school in order to perform a preliminary basic research regarding the role of Vitamin D in neural dopamine metabolism.

## A48 The history of evidence-based medicine: the establishment of scientific medicine, 1815–1875

### John Wallace

#### Institute of Continuing Education, University of Cambridge, Cambridge, United Kingdom

##### **Correspondence:** John Wallace

*Annals of General Psychiatry* 2018, **17(Suppl 1):**A48

**Background:** The first half of the nineteenth century saw a rise in experimental medicine. Scientific discoveries altered the way that medical students were trained and the way that doctors thought about disease and health. Cell theory triumphed, the word ‘scientist’ was coined, and medical advances were accompanied by considerable professional evolution. Education and research became a priority 1.

Trinity College Dublin was founded in 1592 by Elizabeth I of England 2. With the development of a medical school in 1711, ideas and discoveries from Padua and Leiden slowly impacted on the school. However, early nineteenth-century medical science was contested 3. Ideas and discoveries from laboratories and research institutes were resisted. The medical school located in Trinity, with its new scientific ethos, was perceived as foreign and alien.

**Research Aims:** This project will have a number of key foci: the significant impact of Continental medicine on the Dublin medical school; the important scientific innovations introduced from 1813; the resistance of Trinity College to the medical school’s emphasis on anatomical dissection, scientific method, experimental chemistry, and bedside medicine; and the eventual acceptance of the medical school, in 1875, by Trinity College Dublin.

Sources: The primary sources will be accessed in the Manuscripts and Archives Research Library of Trinity College Dublin, the Irish National Archive, the Library of the Royal College of Physicians in Ireland, and the Medical College Library of St. Bartholomew’s Hospital, London. Relevant electronic databases will be accessed, including the Lind Library, The Cochrane Library, and Embase.

**Results:** Significant material will be brought together in a new context and the history of the medical school will provide a way into assessing the impact of science on both medical practice and medical education. During the period under investigation, Trinity began appointing dynamic professors who regularly visited Leiden, Berlin, and Paris. Emphasis was now placed on the utilization of the most recent research and the creation of new knowledge. Discoveries from laboratories and research institutes on the Continent were adopted. Regular autopsies were conducted. Pathology and midwifery were integrated into the curriculum and English, rather than Latin was introduced into the final examinations.

**Conclusion:** The number of students from England and America grew considerably following the introduction of a number of scientific innovations. This research project will establish how medical education and practice became increasingly science-based during the nineteenth century, forming a platform for the dynamic evidence-based movement of the twenty-first century.

1. Porter, R., The greatest benefit to mankind. London: Harper Collins; 1997.

2. Coakley, D., Medicine in Trinity College Dublin. Dublin: Trinity College Dublin; 2014.

3. Bynum, W. F., Science and the practice of medicine in the nineteenth century. Cambridge: Cambridge University Press; 1994.

